# Supplementary material for: Estimating waiting times, patient flow, and waiting room occupancy density as part of tuberculosis infection prevention and control research in South African primary health care clinics
Source: PLOS Glob Public Health. 2022 Jul 20;2(7):e0000684. doi: 10.1371/journal.pgph.0000684 (PMC10021248; doi:10.1371/journal.pgph.0000684)
Supplement: S1 Text — (PDF) [file pgph.0000684.s001.pdf]

SUPPLEMENTARY MATERIAL

Estimating waiting times, patient flow, and waiting room occupancy density as part of tuberculosis infection prevention and control research in South African primary health care clinics

Aaron S Karat[1,2], Nicky McCreesh[1], Kathy Baisley[1,3], Indira Govender[1], Idriss I Kallon[4,5], Karina Kielmann[2], Hayley MacGregor[6], Anna Vassall[1], Tom A Yates[7], Alison D Grant[1,3,8]

1. TB Centre, London School of Hygiene & Tropical Medicine, London, United Kingdom; 2. The Institute for Global Health and Development, Queen Margaret University, Edinburgh, United Kingdom; 3. Africa Health Research Institute, School of Laboratory Medicine & Medical Sciences, College of Health Sciences, University of KwaZulu-Natal, Durban, South Africa; 4. Division of Social and Behavioural Sciences, Faculty of Health Sciences, University of Cape Town, Cape Town, South Africa; 5. Centre for Evidence-based Health Care, Division of Epidemiology and Biostatistics, Department of Global Health, Faculty of Medicine and Health Sciences, Stellenbosch University, Cape Town, South Africa; 6. The Institute of Development Studies, University of Sussex, Brighton, United Kingdom; 7. Department of Infectious Disease, Faculty of Medicine, Imperial College London, London, United Kingdom; 8. School of Public Health, Faculty of Health Sciences, University of the Witwatersrand, Johannesburg, South Africa

Email: aaron.s.karat@gmail.com

Contents

Appendix 1. Literature review to inform choice of method **2**
1.1. Methods 2
1.1.1. Choice of data collection method 2
1.1.2. Searching, sifting, and inclusion and exclusion criteria 2
1.1.3. Data extraction 3
1.2. Results 3
1.2.1. Methods considered; criteria used to make choice 3
Appendix 2. Additional methods **8**
2.1. Data collection 8
2.2. Analysis 10
2.2.1. Multiple imputation 10
Appendix 3. Additional results **13**
3.1. Demographics 13
3.2. Total time spent in clinic 13
3.2.1. Time of arrival 13
3.3. Proportion of time spent indoors vs outdoors 17
3.4. Occupancy density 22
Appendix 4. Additional discussion **23**
Appendix 5. Acknowledgments **24**
Appendix 6. References **27**

Appendix 1. Literature review to inform choice of method

1.1. Methods

1.1.1. Choice of data collection method

A literature review was undertaken to find a data collection method that:

1. Allowed collection of data on the movement of all visitors to the clinic, including those accompanying patients and children, and all clinic staff;
2. Allowed collection of data on movement in all parts of the clinic, including waiting areas;
3. Allowed collection of anonymised data about basic demographics of those attending (age group, sex, reason for attendance) and allowed for identification of those attending with a baby;
4. Did not involve the installation of additional technical (or other) equipment or infrastructure in clinics;
5. Could easily be moved between clinics;
6. Did not violate the privacy or confidentiality of clinic attendees or staff;
7. Was not excessively expensive; and
8. Ideally, could be used (after modification, if needed) in routine practice after the conclusion of the study.

1.1.2. Searching, sifting, and inclusion and exclusion criteria

MEDLINE (via PubMed), Scopus, Web of Science, CINAHL, and Google Scholar, were searched using variations (including Medical Subject Heading [MeSH] terms) and combinations of the following terms: “queue”, “patient flow”, “waiting times”, “measurement”, “modelling”, “lean”, and “six sigma”. Results from the different databases were compared and duplicate records removed. Titles and abstracts were then hand-sifted; studies were included that described measurement of the physical movement of individuals through a defined structure or system (including, but not limited to emergency departments, hospitals, airports, and retail outlets); reported on the measurement of the movement of other objects through a defined structure or system (including reports of manufacturing processes and applications of traffic flow theory); attempted to measure the size and/or density of crowds of people, either indoors or outdoors; and reported on methods used to analyse similar data (including applications of queue theory). Articles were excluded that described the application of queue theory to the design or implementation of computing networks or systems (for example, studies of methods to improve management of web traffic) or that were purely theoretical (i.e., that did not describe the collection or analysis of data).

1.1.3. Data extraction

Data were extracted by one individual; for articles reporting on data collection, information captured include the study setting, the objectives of the data collection exercise, the outcomes of interest, and a detailed description of the methods used to collect data. For studies reporting on analyses of previously collected data, information captured included any available details of how the data were collected, the outcomes of interest, a description of the analysis process, and any theory used to inform it. A pragmatic, snowballing approach was adopted: where an article described a method that was the same as or very similar to one that had already been documented, only major differences in setting and methods were recorded, instead of all the details described above. As this was a review of methods, findings from included studies were not extracted.

Records were organised using Mendeley and Microsoft Excel; Microsoft Access was used for record sifting and data extraction.

1.2. Results

1.2.1. Methods considered; criteria used to make choice

Table A summarises some of the methods that have been used to measure waiting times and patient flow in health care settings and Table B provides a summary of methods identified and the dimensions they could be used to measure.

Table A. Overview of methods used in previous studies (assigned to broad categories)

#	Category	Description/elements	Key references/examples
**1**	**Paper-based systems**	1. Log times of patient entry and exit from the clinic<br>2. Simple, often paper-based system for tracking patients' movement around clinics (allows for estimation of time at a particular station as well as time spent between stations/in waiting rooms)<br>3. Observation of patient flow by research team<br>4. Direct observation/measurement of consultation times with various groups of HCW	Bachman 1997[1]<br>Ideal clinic[2]<br>Reagon 2010[3]
**2**	**Real-time location systems**	e.g., Radiofrequency identification systems used to track waiting times/patient movement within a facility	Singman 2015[4]
**3**	**Proximity sensors**	'Protractor' – uses infra-red light to measure distance and relative body orientation of interacting users	Montanari 2018[5]
		Social fMRI - includes a mobile (android-based) programme that records proximity to other phones	Aharony 2011[6]
		iEpi – an application that collects data on social interactions (but only between people that have the application)	Aiello 2016[7]
		Other, low-tech ways to estimate proximity/interaction (e.g., interviews, observation, diaries, etc.)	
**4**	**Systems for estimating queue sizes & durations**	Used widely in commercial settings: airports, retail; often involve infra-red technology/use of other sensors; use of proprietary software	https://www.airport-suppliers.com/supplier/qmetrix-gmbh/
**5**	**Camera-based systems (video or still images)**	Used in retail; use 'video management system' as a baseline – can then provide information on ingress, egress, queue length, waiting time<br>Various levels of sophistication – can use machine learning/AI, face recognition; needs fairly heavy-duty computing infrastructure (1TB HDD server	www.allgovision.com
		**Data-driven crowd analysis** – software that analyses video images of crowds and 'learns' patterns by performing long-term analysis in an off-line manner.<br>"…learn crowd motion patterns by performing long-term analysis in an off-line manner. The learned motion patterns can be used in a range of application domains such as crowd event detection or anomalous behaviour recognition. …The idea is that any given crowd video can be thought of as being a mixture of previously observed videos."	Rodriguez 2011[8]
		**Density-aware tracking**<br>"Detecting and tracking people in crowded scenes is a crucial component for a wide range of applications including surveillance, group behaviour modelling and crowd disaster prevention. The reliable person	Rodriguez 2011[9]

#	Category	Description/elements	Key references/examples
		detection and tracking in crowds, however, is a highly challenging task due to heavy occlusions, view variations and varying density of people as well as the ambiguous appearance of body parts, e.g. the head of one person could be similar to a shoulder of a near-by person. High-density crowds present particular challenges due to the difficulty of isolating individual people with standard low-level methods of background subtraction and motion segmentation typically applied in low-density surveillance scenes."	
		**Structured vs. unstructured crowd tracking**<br>"In an unstructured crowded scene, the motion of the crowd appears to be random, with different participants moving in different directions at different times. That is, in such scenes each spatial location supports more than one, or multi-modal, crowd behaviour. For instance, a video of people walking on a zebra-crossing in opposite directions is an example of an unstructured crowded scene because, broadly speaking, at any point on the zebra crossing the probability of observing a person moving from left to right is as likely as observing a person walking from right to left. Other examples of such scenes include exhibitions, crowds in a sporting event, railway stations, airports, and motion of biological cells."	Rodriguez 2009[10]
**6**	**Measurement of structured or linear flow (e.g., traffic)**	**Measurements at a point or between two points**<br>• "This method is easily capable of providing volume counts and therefore flow rates directly, and with care can also provide time headways"	Traffic flow theory[11]
		**Measurements over a short section**<br>• "All of these presence detectors continue to provide direct measurement of volume and of time headways, as well as of speed when pairs of them are used"	
		**Measurements using (a) moving observer/s**<br>• "While the intention in this method is that the floating car behaves as an average vehicle within the traffic stream, the method cannot give precise average speed data. It is, however, effective for obtaining qualitative information about freeway operations without the need for elaborate equipment or procedures."	
		**Measurements across a system/wide area**<br>• The limitation to all three systems is that they can realistically be expected to provide information only on speeds. It is not generally possible for one moving vehicle to be able to identify flow rates or densities in any meaningful way."	
**7**	**Broader/ systemic/ approaches to flow/ queue management**	**Queue science**<br>• "Patient flow in hospitals can be naturally modelled as a queueing network, where patients are the customers, and medical staff, beds and equipment are the servers.")	Armony 2015[12]

#	Category	Description/elements	Key references/examples
		**Operations research/management**<br>• "Having time varying arrivals and heterogeneous patients that need to be treated in consecutive processing steps by several doctors, nurses and other employees, it is a complex environment to control."<br>• "Measurement of crowding: A fundamental weakness is the lack of a measurement gold standard. There is a weak literature base describing scoring systems of crowding."	Carmen 2014[13]<br>Higginson 2012[14]
		**Various QI approaches**<br>• Linear process evaluation<br>• Action planning<br>• Patient flow simulator	Bean 2019;[15] Patient flow simulator: https://khp-informatics.github.io/patient-flow-simulator/
		**Game theoretical approaches**<br>• Patient satisfaction	McAdams 2014[16]

Table B. Overlap between outcomes of interest and methods of measurement identified through literature review

**Outcomes**	**Numbers of people**		**Time**				**Proximity/density**			**Movement**	
**Method of measurement**	Numbers in and out of the clinic	Numbers in and out of different parts of the clinic	'Average' time spent by individuals in the facility	Time spent by specific individuals in the facility	Time spent by specific individuals in different parts of the clinic	Time spent in proximity with other people/ other specific people	How/ how much people interact with each other	How closely people are grouped	Coughing/ other proxies for transmission	Average speed between key points/ through a particular area	Overall efficiency of the clinic
Motion sensors/footfall cameras/counting numbers in & out	**X**	**X**	**X**							**(X)**	
**Waiting/working time survey (paper-based or real-time location system)**	**X**	**X**	**X**	**X**	**X**	**(X)**			**(X)**	**X**	**(X)**
Proximity sensors							**X**	**X**	**(X)**		
Cough sensors									**X**		
Systems for estimating queue lengths (numeric size and duration)					**X**					**X**	**(X)**
Camera-based systems	**X**	**X**	**X**	**(X)**	**(X)**		**X**	**X**	**(X)**		
Measurement of structured or linear flow (e.g., traffic)			**X**							**X**	**(X)**
Queue science approaches	**(X)**	**(X)**								**X**	**(X)**
Operations research/management approaches	**(X)**		**(X)**								**X**
QI approaches	**(X)**		**(X)**				**(X)**				**X**

x = reliably estimated; **(x)** = partially estimated

Appendix 2. Additional methods

2.1. Data collection

Figure A. Flow diagram illustrating data collection process.

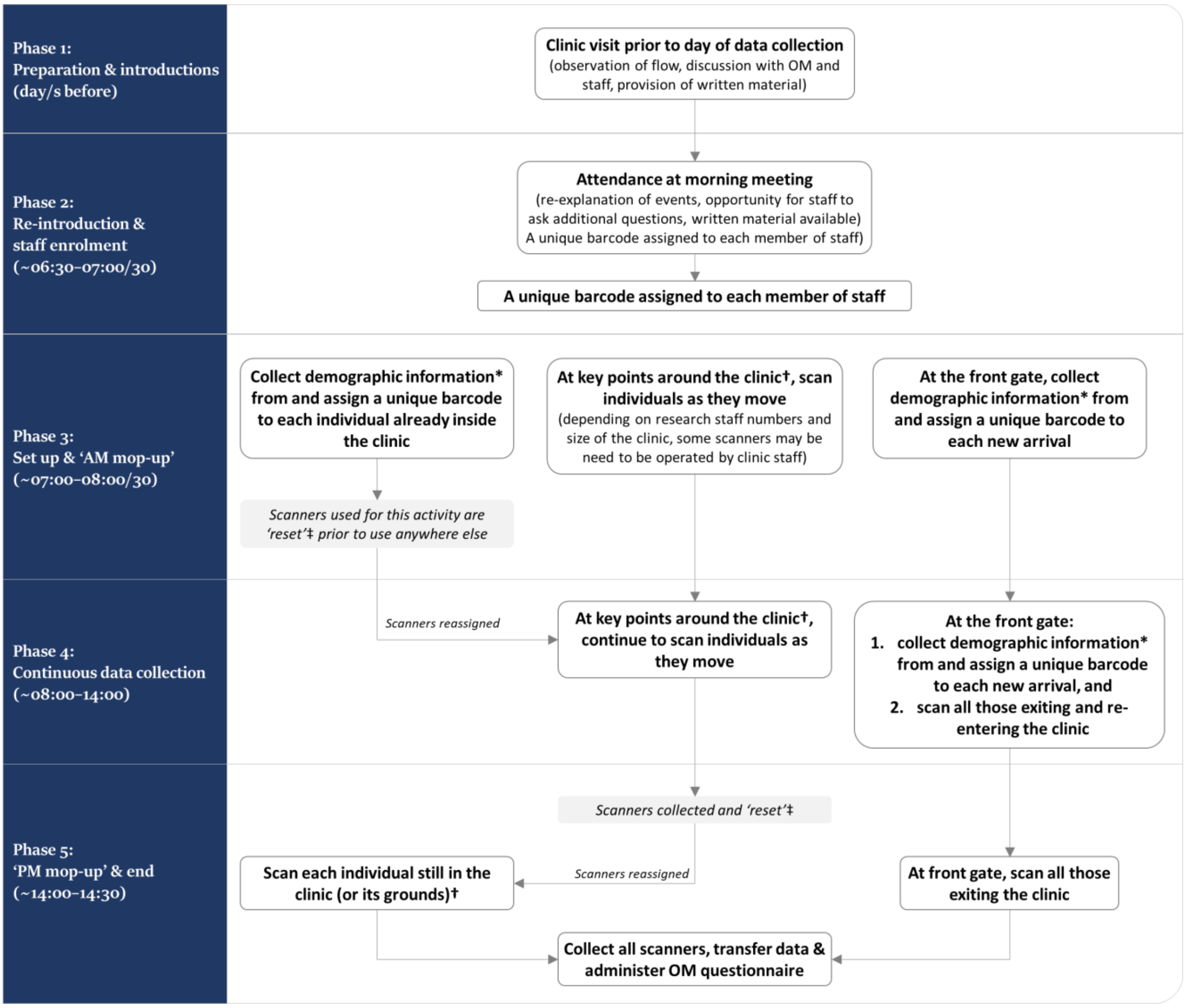

*Only non-identifiable information collected; participants were free to decline to participate
†Scanners were numbered and were 'location specific'. A record was kept of which scanner was used where.
‡A designated barcode was scanned three times in quick succession to denote reassignment of scanner. Time of reset was documented as well as original and new locations of the scanner.

Figure B. Cards used for data collection from patients and clinic visitors (left) and from clinic staff (right)

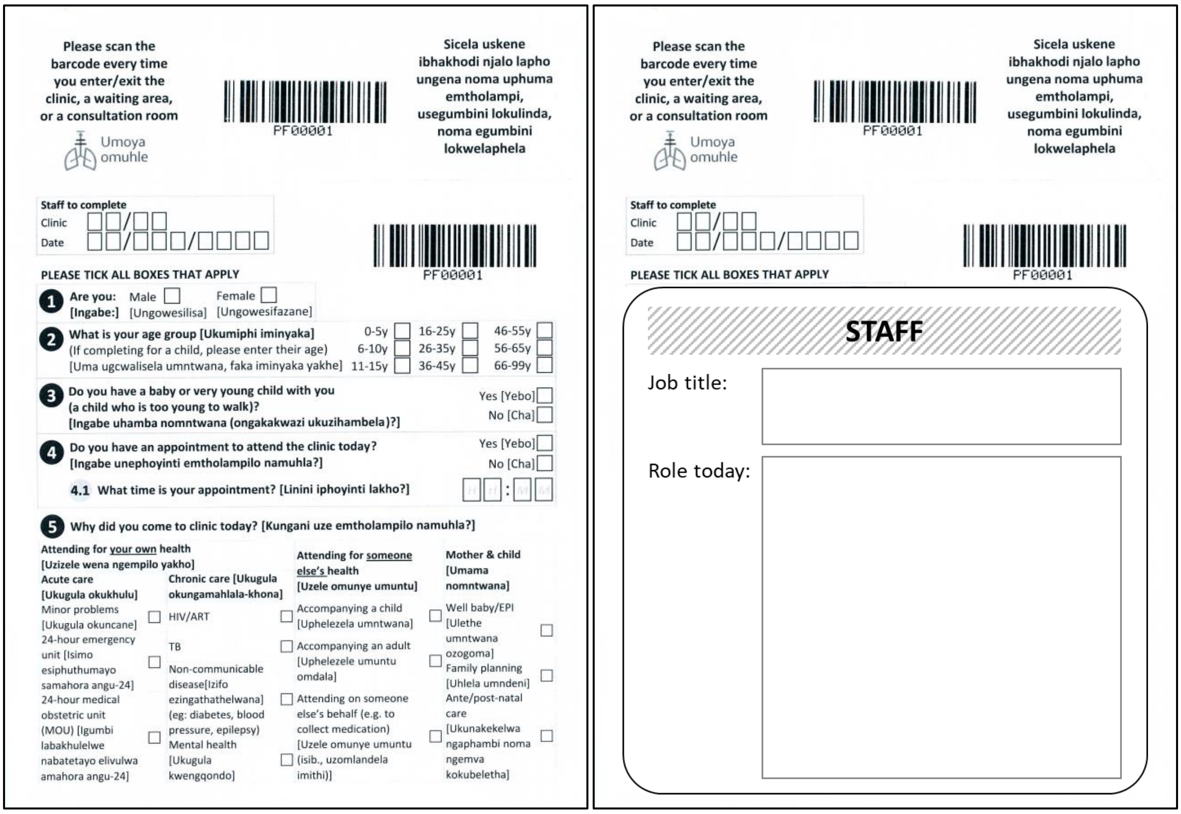

Figure C. Schematic of an imagined small clinic, showing positioning of barcode scanners held by researchers and clinic staff

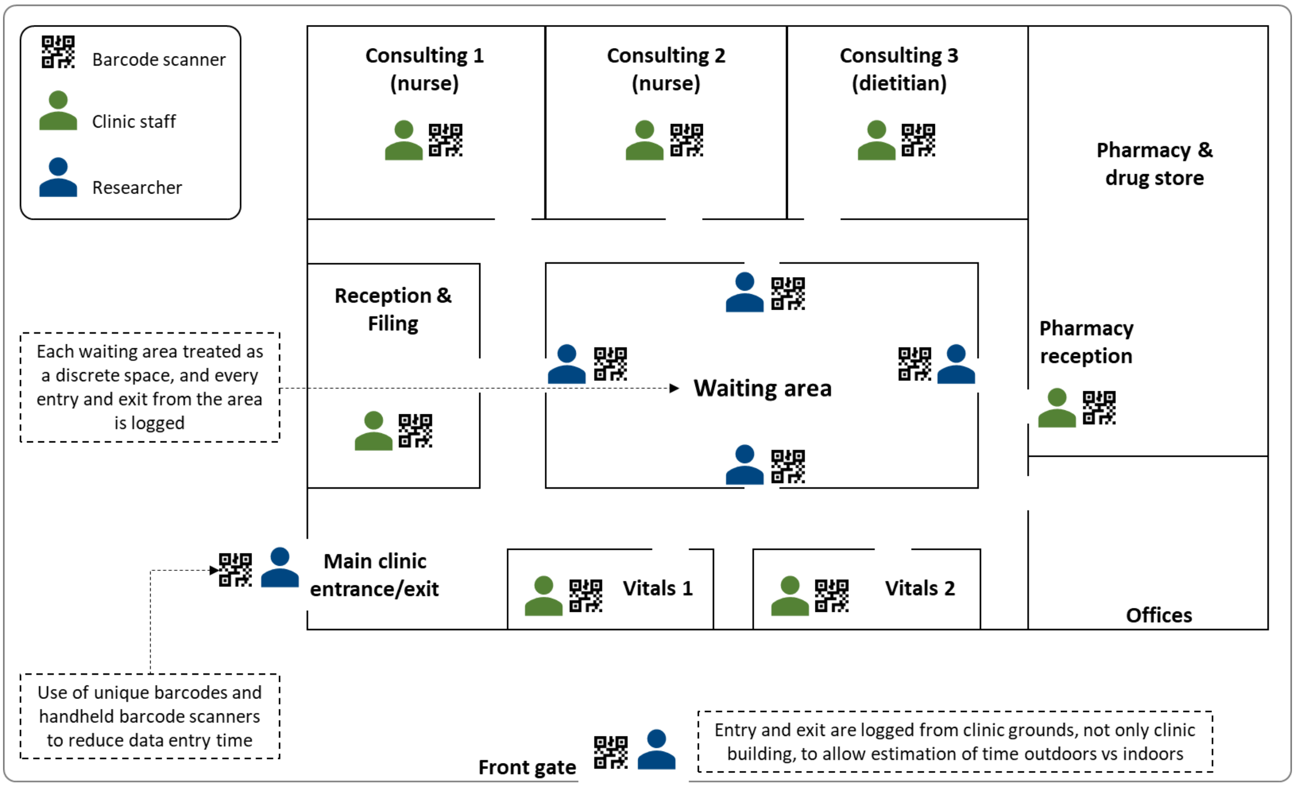

2.2. Analysis

Table C. Number of clinics, number of individuals, and type of data used for each stage of the analysis

Analysis	Data used	Number of clinics	Clinics included	Number of data collection exercises	Additional exclusion criteria	Individuals included in analysis
Demographics	Original	11	KZN1, KZN2, KZN3, KZN4, KZN5, KZN6, WC1, WC2, WC3, WC4, WC5, WC6	12*	None	2,903
Time spent in clinic†	Imputed	10	KZN1, KZN2, KZN3, KZN5, KZN6, WC1, WC2, WC3, WC4, WC5, WC6	11*	None	2,634
Proportion of time spent indoors vs. outdoors‡	Original	9	KZN1, KZN2, KZN3, KZN6, WC1, WC2, WC3, WC4, WC5, WC6	10*	Total visit time <5 minutes and no recorded exit	2,190
Occupancy density of indoor spaces§	Original	3	KZN2, KZN6, WC1	3	None	847

*Clinic KZN1 visited twice.
†Clinic KZN4 excluded as entry and exit from clinic not captured.
‡Clinics KZN4 and KZN5 excluded as coverage of all gates and indoor/outdoor doors not achieved.
§Suitable data only available from three clinics with multiple indoor waiting areas.
KZN: KwaZulu-Natal; WC: Western Cape

2.2.1. Multiple imputation

Four key times were identified in the pathways that each clinic attendee took through the clinic.

1. **Time of arrival**

   The time that they first arrived at the clinic. This was assumed to be the time that their barcode was first scanned, for attendees who arrived after the start of data collection. The arrival time was set to missing if the attendee was already present in the clinic before the start of data collection, or if the first time their barcode was scanned was not at a clinic entrance (an external door or compound gate).

2. **Time at files**

   The time that the attendee obtained their patient file from the clinic reception desk. This was assumed to be the time that their barcode was first scanned at files, provided that it occurred before the first time that they were scanned at vitals or at a consultation room. The time was set to missing if they never scanned at files, or if they scanned at vitals or a consultation room before first scanning at files.

3. **Time at vitals**

   The time that the attendee has their blood pressure, heart rate, and respiratory rate measured. This was assumed to be the time that their barcode was first scanned at vitals, provided that it occurred before the first time that they were scanned at a consultation room. The time was set to missing if they never scanned at vitals, or if they scanned at a consultation room before first scanning at vitals.

4. **Time of departure**

   The time that the attendee left the clinic. This was assumed to have occurred at the final time that they scanned their barcode, provided it occurred at a clinic exit point (an external door or compound gate). The leaving time was set to missing for attendees who were still at the clinic at the end of data collection, or if their barcode was never scanned at an exit point.

In a small number of cases, times at files and/or vitals were missing not because the attendee did not scan their barcode, but because the attendee did not complete that stage. For instance, some attendees who were at the clinic to collect medicine only may have skipped one or both stages. In many clinics, patients on TB treatment also skip the files and vitals stages. In all ten clinics however, the majority of patients were required to pass through both files and vitals, regardless of their visit reason.

Missing times (Table D) were imputed as interval-censored values, with lower and upper bounds of when the event would have occurred, using a sequential approach. First, arrival times at the clinic were multiply-imputed using 20 imputations. For attendees who arrived before the start of data collection, the lower and upper limits of the time of arrival were set to the clinic opening time and the start of data collection, respectively. For those who arrived after the start, the lower limit was set as the start of data collection, and the upper limit was the time that the attendee was first scanned. Second, the time at files was imputed, using the imputed arrival time as the lower bound of the interval and time at vitals (if observed) as the upper limit. If time at vitals was not observed, the upper bound was set to the earliest of the maximum time from arrival to files observed in that clinic, the time of leaving (if observed), the end of data collection (if not there at end) or, close of clinic (if there at end). Third, the time at vitals was imputed, using the imputed time at files as the lower bound of the interval, and the setting the upper bound to the earliest of the maximum time from files to vitals observed in that clinic, the time of leaving (if observed), the end of data collection (if not there at end), or the close of clinic (if there at end). Finally, the time of leaving the clinic was imputed, using the imputed time at vitals as the lower bound, and setting the upper bound to the earliest of the maximum time from vitals to leaving observed in that clinic, end of data collection (if not there at end) or close of clinic (if there at end).

Table D. Number of attendees and number with data missing for time of arrival, files, vitals, and departure at each clinic from which data underwent multiple imputation (n = 2,634)

Clinic code	Attendees, n	Arrival, n (%/attendees)			Files, n (%/attendees)		Vitals, n (%/attendees)		Departure, n (%/attendees)		
		Known	Missing (arrived early)‡	Missing (other)	Known	Missing	Known	Missing	Known	Missing (left late)§	Missing (other)
KZN1 (#1)*	234	77 (33)	10 (4)	147 (63)	87 (37)	147 (63)	61 (26)	173 (74)	91 (39)	70 (30)	73 (31)
KZN1 (#2)†	417	264 (63)	135 (32)	18 (4)	146 (35)	271 (65)	155 (37)	262 (63)	248 (59)	129 (31)	40 (10)
KZN2	170	130 (76)	36 (21)	4 (2)	64 (38)	106 (62)	81 (48)	89 (52)	120 (71)	47 (28)	3 (2)
KZN3	270	183 (68)	78 (29)	9 (3)	10 (4)	260 (96)	77 (29)	193 (71)	220 (81)	34 (13)	16 (6)
KZN5	347	241 (69)	84 (24)	22 (6)	18 (5)	329 (95)	38 (11)	309 (89)	246 (71)	89 (26)	12 (3)
KZN6	218	128 (59)	63 (29)	27 (12)	109 (50)	109 (50)	120 (55)	98 (45)	174 (80)	34 (16)	10 (5)
WC1	337	224 (66)	65 (19)	48 (14)	0	337 (100)	133 (39)	204 (61)	194 (58)	79 (23)	64 (19)
WC2	69	65 (94)	2 (3)	2 (3)	52 (75)	17 (25)	31 (45)	38 (55)	56 (81)	11 (16)	2 (3)
WC3	120	56 (47)	44 (37)	20 (17)	51 (43)	69 (58)	41 (34)	79 (66)	54 (45)	38 (32)	28 (23)
WC5	308	110 (36)	158 (51)	40 (13)	33 (11)	275 (89)	27 (9)	281 (91)	176 (57)	43 (14)	89 (29)
WC6	144	93 (65)	40 (28)	11 (8)	54 (38)	90 (63)	70 (49)	74 (51)	121 (84)	17 (12)	6 (4)
**Total**	**2,634**	**1,571 (60)**	**715 (27)**	**348 (13)**	**624 (24)**	**2,010 (76)**	**834 (32)**	**1,800 (68)**	**1,700 (65)**	**591 (22)**	**343 (13)**

*First data collection exercise
†Second data collection exercise
‡Arrived before the start of data collection.
§Left after the end of data collection

Age, sex, clinic, reason for visit, whether there at start/end, and whether the attendee was first scanned in the morning (before 10am) were included in the imputation model. Twenty imputed datasets were created.

Appendix 3. Additional results

3.1. Demographics

Table E. Characteristics of primary health care clinics at which patient flow data collection exercises were conducted (n = 11)

Clinic code	Decade built	Location	PHC clinic or CHC	Estimated monthly head count, thousands	Date-time appointment system?*	Covered outdoor waiting area used as part of patient pathway?	Data collection exercises, n	Number of attendees included per exercise
KZN1	1990s	Semi-rural	PHC	11–14	No	Yes†	2	Exercise 1: 234; exercise 2: 417
KZN2	1980s	Rural	PHC	5–8	No	Yes	1	170
KZN3	2000s	Peri-urban	CHC	27–30	No	Yes	1	270
KZN4	1980s	Urban	CHC	27–30	No	Yes	1	269§
KZN5	1980s	Urban	PHC	4–7	No	Yes	1	347
KZN6	2000s	Rural	PHC	3–6	No	No‡	1	218
WC1	2010s	Peri-urban	PHC	25–28	Yes	No	1	337
WC2	2000s	Urban	PHC	1–3	Yes	No	1	69
WC3	1980s	Peri-urban	PHC	2–5	Yes	No	1	120
WC5	2010s	Urban	CHC	27–30	Yes	No‡	1	308
WC6‖	2000s	Peri-urban	PHC	25–28	Yes	No	1	144

*Often only for patients attending for selected services
†Used only for selected patients (‘chronic stream’)
‡Outdoor area not part of normal patient pathway; used primarily before the clinic opens
§Data collected from HIV/chronic unit only
‖Not officially a CHC but as large as and with an equivalent patient load to most CHCs.
CHC: community health centre; PHC: primary health care; KZN: KwaZulu-Natal; WC: Western Cape

3.2. Total time spent in clinic

3.2.1. Time of arrival

Most individuals arrived early: overall median time of arrival was 09:00 (IQR 07:53–10:27. This was similar across all clinics (range 07:36–09:36) and between provinces (Figure D).

Figure D. Histograms showing distribution of imputed time of arrival at clinic by province (n = 2634; 20 imputations)

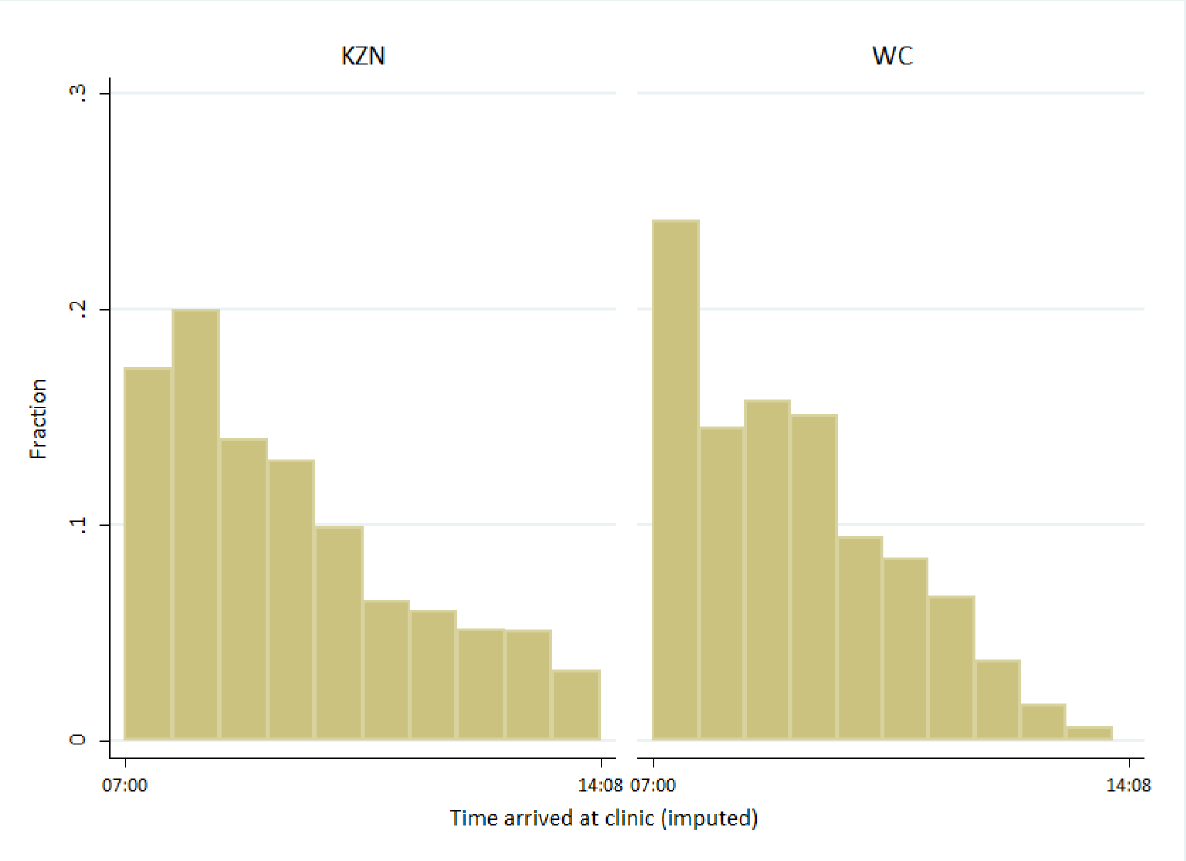

The shape of the relationship between time of arrival and time spent in clinic was examined using fractional polynomials, after extraction of one imputed dataset. The optimal second degree fractional polynomial of arrival time had the terms arrival time$^{(-2, 3)}$. However, there was no evidence that the optimal second degree or first degree fractional polynomial fit the data better than the linear model (p = 0.646 and p = 0.436, respectively) and time of arrival was included into the final model as a linear term.

Table F. Total time spent in clinic, by province and demographics (generated using imputed data; n = 2,634)

Demographic	Overall (n = 2,634)		KwaZulu-Natal (n = 1,656)		Western Cape (n = 978)	
	n (%)	Median (IQR) [range], HH:MM	n (%)	Median (IQR) [range] , HH:MM	n (%)	Median (IQR) [range], HH:MM
**Overall**	2,634 (100)	02:36 (01:36–03:43) [00:06–10:01]	1,656 (100)	02:33 (01:35–03:40) [00:09–10:01]	978 (100)	02:42 (01:37–03:49) [00:06–08:09]
**Sex**						
Male	783 (29.7)	02:19 (01:25–03:32) [00:09–09:44]	467 (28.2)	02:16 (01:27–03:24) [00:11–09:44]	316 (32.3)	02:26 (01:21–03:39) [00:09–08:09]
Female	1,851 (70.3)	02:42 (01:41–03:47) [00:06–10:01]	1,189 (71.8)	02:38 (01:38–03:44) [00:09–10:01]	662 (67.7)	02:48 (01:46–03:55) [00:06–08:00]
**Age group**						
<16 years	381 (14.5)	02:50 (01:45–03:48) [00:09–10:01]	227 (13.7)	02:45 (01:42–03:46) [00:09–10:01]	154 (15.7)	02:55 (01:51–03:48) [00:10–06:44]
16–45 years	1,703 (64.7)	02:34 (01:36–03:43) [00:09–08:53]	1,113 (67.2)	02:30 (01:34–03:37) [00:10–08:53]	590 (60.3)	02:43 (01:41–03:53) [00:09–08:09]
≥46 years	550 (20.9)	02:33 (01:32–03:40) [00:06–08:25]	316 (19.1)	02:34 (01:34–03:43) [00:16–08:25]	234 (23.9)	02:32 (01:26–03:36) [00:06–07:47]
**Carrying a baby or very young child**						
No	2,271 (86.5)	02:32 (01:34–03:40) [00:06–10:01]	1,465 (88.6)	02:29 (01:33–03:36) [00:09–10:01]	806 (82.8)	02:39 (01:36–03:48) [00:06–08:09]
Yes	344 (13.1)	03:04 (01:51–04:07) [00:18–08:21]	187 (11.3)	03:09 (01:59–04:22) [00:29–08:21]	157 (16.1)	02:55 (01:42–03:58) [00:18–07:15]
NR	12 (0.5)	03:10 (01:47–04:02) [00:49–06:42]	4 (0.1)	01:47 (01:47–01:47) [01:47–01:47]	11 (1.1)	03:20 (01:58–04:02) [00:49–06:42]
**Attending with ≥1 other person**						
No	1,983 (75.3)	02:31 (01:33–03:39) [00:06–08:53]	1,279 (77.2)	02:28 (01:32–03:35) [00:10–08:53]	704 (72.0)	02:38 (01:37–03:44) [00:06–08:09]
Yes	651 (24.7)	02:53 (01:44–03:57) [00:09–10:01]	377 (22.8)	02:50 (01:46–03:53) [00:09–10:01]	274 (28.0)	02:57 (01:36–03:58) [00:10–08:00]
**Time of arrival**						
0700–0759	737 (28.0)*	03:08 (02:06–04:19) [00:23–09:44]	443 (26.7)*	03:09 (02:05–04:23) [00:30–09:44]	295 (30.1)*	03:07 (02:06–04:14) [00:23–08:09]
0800–0859	582 (22.1)*	02:40 (01:41–04:03) [00:10–10:01]	358 (21.6)*	02:34 (01:43–03:56) [00:12–10:01]	224 (22.9)*	02:49 (01:36–04:13) [00:10–07:28]
0900–0959	494 (18.7)*	02:38 (01:28–03:42) [00:09–07:18]	299 (18.0)*	02:41 (01:35–03:44) [00:09–07:18]	195 (19.9)*	02:32 (01:19–03:35) [00:09–06:49]
1000–1059	320 (12.2)*	02:38 (01:50–03:28) [00:13–07:30]	195 (11.8)*	02:36 (01:43–03:27) [00:13–07:30]	126 (12.8)*	02:41 (01:52–03:30) [00:30–05:51]
1100–1159	234 (8.9)*	02:06 (01:19–02:57) [00:06–07:08]	141 (8.5)*	02:06 (01:16–02:59) [00:12–07:08]	93 (9.5)*	02:06 (01:25–02:57) [00:06–05:23]
1200–1259	152 (5.8)*	01:30 (00:59–02:30) [00:13–06:04]	116 (7.0)*	01:34 (01:05–02:36) [00:14–06:04]	36 (3.7)*	01:18 (00:53–02:04) [00:13–04:12]
≥1300	115 (4.4)*	01:46 (01:02–02:28) [00:10–06:03]	105 (6.3)*	01:47 (01:03–02:27) [00:10–05:59]	10 (1.0)*	01:43 (00:55–02:42) [00:10–06:03]

**Demographic**	**Overall (n = 2,634)**		**KwaZulu-Natal (n = 1,656)**		**Western Cape (n = 978)**	
	n (%)	Median (IQR) [range], HH:MM	n (%)	Median (IQR) [range] , HH:MM	n (%)	Median (IQR) [range], HH:MM
**Reported main reason for visit**						
Acute care/HIV care	1,526 (57.9)	02:37 (01:39–03:45) [00:09–10:01]	1,008 (60.9)	02:30 (01:36–03:38) [00:09–10:01]	518 (53.0)	02:50 (01:48–03:57) [00:10–08:09]
Tuberculosis	145 (5.5)	02:00 (01:08–02:55) [00:09–07:36]	78 (4.7)	01:48 (01:04–02:43) [00:11–07:36]	67 (6.9)	02:12 (01:12–03:09) [00:09–06:37]
NCDs	157 (6.0)	02:34 (01:31–03:45) [00:10–07:40]	63 (3.8)	02:28 (01:27–03:45) [00:19–07:40]	94 (9.6)	02:39 (01:33–03:48) [00:10–06:57]
Mother & child	297 (11.3)	02:47 (01:41–03:43) [00:22–08:19]	214 (12.9)	02:57 (01:51–03:47) [00:22–08:19]	83 (8.5)	02:23 (01:21–03:27) [00:39–06:50]
Ante/post-natal	66 (2.5)	02:33 (01:32–03:25) [00:12–05:57]	42 (2.5)	02:30 (01:29–03:07) [00:12–05:57]	24 (2.5)	02:43 (01:40–03:30) [00:28–05:28]
Accompanying	360 (13.7)	02:45 (01:34–03:58) [00:06–09:28]	202 (12.2)	02:42 (01:36–03:58) [00:12–09:28]	158 (16.2)	02:51 (01:32–03:57) [00:06–06:55]
Proxy	79 (3.0)	02:18 (01:22–03:28) [00:11–08:19]	47 (2.8)	02:41 (01:33–03:49) [00:11–08:19]	32 (3.3)	01:59 (00:58–02:53) [00:23–06:49]
NR	4 (0.2)	03:50 (03:12–04:39) [01:24–05:57]	2 (0.1)	03:58 (03:14–04:40) [03:05–04:45]	2 (0.2)	03:50 (03:01–04:23) [01:24–05:57]

*Mean of 20 imputations

HH: hours; IQR: interquartile range; MM: minutes; NCD: non-communicable disease; NR: not recorded;

3.3. Proportion of time spent indoors vs outdoors

Table G. Proportion of captured time at clinic spent indoors vs. outdoors (n = 2,190 individuals with total time captured of five minutes or longer; n = 10 visits to 9 clinics)

Clinic code	≥1 outdoor waiting area?	Individuals captured, n	Time captured per individual (minutes), median (IQR) [range]	Proportion of time spent indoors (%), median (IQR) [range]	Proportion of captured time spent outdoors (%), median (IQR) [range]	Proportion of captured time spent in unknown location (%), median (IQR) [range]
**All clinics**		**2,190**	**120.8 (58.3–205.9) [5.1–404.3]**	**95.6 (45.6–100) [0–100]**	**2.5 (0–35.3) [0–100]**	**0 (0–0) [0–100]**
Clinics with ≥1 outdoor waiting area		1,362	126.6 (62.0–126.6) [5.1–404.3]	73.7 (13.6–97.8) [0–100]	13.7 (1.4–74.5) [0–100]	0 (0–0) [0–100]
Clinics without an outdoor waiting area		828	115.2 (51.2–193.7) [5.7–381.6]	100 (97.1–100) [0–100]	0 (0–1.4) [0–100]	0 (0–0) [0–100]
**Individual clinics**						
KZN1 (visit 1)	Yes	211	109.5 (53.1–199.8) [6.5–378.5]	91.2 (0–99.3) [0–100]	4.6 (0.5–100) [0–100]	0 (0–0) [0–100]
KZN1 (visit 2)	Yes	380	163.6 (82.8–291.3) [5.1–404.3]	26.1 (0.8–96.6) [0–100]	47.2 (2.0–98.7) [0–100]	0 (0–0) [0–100]
KZN2	Yes	167	161.6 (67.8–217.5) [5.8–352.9]	56.7 (32.5–82.4) [0–99.4]	36.2 (9.8–65.0) [0–100]	0 (0–1.0) [0–97.7]
KZN4	Yes	262	106.7 (58.5–146.9) [5.6–333.1]	85.6 (50.4–100) [0–100]	9.4 (0–35.4) [0–99.2]	0 (0–0) [0–100]
KZN5	Yes	342	132.8 (58.4–203.5) [6.8–370.9]	86.2 (19.2–97.2) [0–100]	5.2 (1.7–40.8) [0–100]	0 (0–3.4) [0–99.7]
KZN6	No	209	85.5 (47.3–132.0) [5.8–323.1]	97.9 (93.8–99.0) [0–100]	1.8 (0.8–5.4) [0–100]	0 (0–0) [0–66.7]
WC1	No	307	145.9 (63.0–230.3) [6.0–381.6]	100 (99.7–100) [0–100]	0 (0–0) [0–100]	0 (0–0) [0–100]
WC2	No	64	124.4 (63.8–193.7) [14.0–363.6]	98.3 (88.0–99.5) [2.6–99.9]	0.8 (0.4–3.4) [0–37.6]	0 (0–1.1) [0–94.3]
WC3	No	111	154.6 (60.3–210.1) [5.8–323.5]	100 (99.2–100) [33.7–100]	0 (0–0.6) [0–56.4]	0 (0–0) [0–66.3]
WC6	No	137	71.1 (24.2–197.2) [5.7–361.1]	100 (100–100) [22.7–100]	0 (0–0) [0–77.3]	0 (0–0) [0–0]

IQR: interquartile range; KZN: KwaZulu-Natal; WC: Western Cape

Table H. Proportion of captured time at clinic spent indoors vs. outdoors, by self-reported reason for attendance (n = 2,190 individuals with total time captured of five minutes or longer; n = 10 visits to 9 clinics)

Clinic code, reason for visit	≥1 outdoor waiting area?	Individuals captured, n	Time captured per individual (minutes), median (IQR) [range]	Proportion of time spent indoors (%), median (IQR) [range]	Proportion of captured time spent outdoors (%), median (IQR) [range]	Proportion of captured time spent in unknown location (%), median (IQR) [range]
**KZN1 (visit 1)**	**Yes**	**211**	**109.5 (53.1–199.8) [6.5–378.5]**	**91.2 (0–99.3) [0–100]**	**4.6 (0.5–100) [0–100]**	**0 (0–0) [0–100]**
Acute care		89	106.7 (56.4–196.7) [7.8–367.5]	96.7 (59.7–99.6) [0–100]	2.9 (0.2–40.3) [0–100]	0 (0–0) [0–100]
HIV/ART		38	91.2 (42.1–151.9) [7.9–344.9]	0 (0–7.4) [0–100]	100 (92.6–100) [0–100]	0 (0–0) [0–81.4]
Other chronic		10	35.1 (18.5–87.0) [16.0–256.7]	0 (0–0) [0–100]	100 (100–100) [0–100]	0 (0–0) [0–0]
TB		2	46.6 (21.9–71.4) [21.9–71.4]	0 (0–0) [0–0]	100 (100–100) [100–100]	0 (0–0) [0–0]
Accompanying a child		22	208.7 (153.3–295.4) [54.0–378.5]	98.4 (80.3–99.8) [3.4–100]	1.6 (0.2–19.7) [0–96.6]	0 (0–0) [0–0]
Accompanying an adult		15	66.1 (43.1–120.8) [15.9–199.8]	95.3 (0–98.3) [0–99.4]	2.0 (0.8–100) [0–100]	0 (0–0) [0–85.7]
Attending for someone else		9	64.4 (36.2–103.5) [6.5–368.6]	0 (0–99.3) [0–99.8]	100 (0.7–100) [0.2–100]	0 (0–0) [0–0]
Mother & child		26	191.0 (63.2–289.6) [33.0–377.5]	98.1 (66.1–100) [15.5–100]	0.9 (0–2.9) [0–66.1]	0 (0–0) [0–84.5]
**KZN1 (visit 2)**	**Yes**	**380**	**163.6 (82.8–291.3) [5.1–404.3]**	**26.1 (0.8–96.6) [0–100]**	**47.2 (2.0–98.7) [0–100]**	**0 (0–0) [0–100]**
Acute care		118	138.8 (80.3–277.6) [5.3–403.4]	89.8 (18.9–98.3) [0–100]	7.2 (1.0–50.1) [0–100]	0 (0–0) [0–100]
HIV/ART		125	144.6 (64.0–294.8) [6.9–404.3]	1.3 (0–6.4) [0–96.4]	98.6 (92.8–100) [0–100]	0 (0–0) [0–10.4]
Other chronic		18	206.0 (103.8–264.9) [10.3–397.9]	0.1 (0–1.6) [0–10.4]	99.9 (98.4–100) [89.6–100]	0 (0–0) [0–0]
TB		3	179.3 (49.2–391.7) [49.2–391.7]	35.8 (0.3–42.8) [0.3–42.8]	64.2 (57.2–99.7) [57.2–99.7]	0 (0–0) [0–0]
Accompanying a child		43	257.1 (165.3–358.6) [14.3–402.7]	97.5 (81.4–99.3) [0–100]	2.0 (0.5–6.6) [0–100]	0 (0–0.3) [0–88.7]
Accompanying an adult		18	102.3 (60–268.3) [5.8–393.7]	90.4 (4.7–98.0) [0–100]	5.5 (1.4–53.1) [0–100]	0 (0–0) [0–100]
Attending for someone else		11	150.4 (85.1–344.1) [37.1–392.7]	8.2 (0–35.7) [0–95.1]	91.8 (64.3–100) [4.1–100]	0 (0–0) [0–0.9]
Mother & child		42	153.6 (84.2–277.7) [5.1–394.3]	96.7 (68.6–99.4) [0–100]	1.0 (0.3–5.7) [0–51.8]	0 (0–1.1) [0–100]
Not recorded		2	223.1 (177.9–268.3) [177.9–268.3]	51.1 (2.4–99.7) [2.4–99.7]	48.9 (0.3–97.6) [0.3–97.6]	0 (0–0) [0–0]
**KZN2**	**Yes**	**167**	**161.6 (67.8–217.5) [5.8–352.9]**	**56.7 (32.5–82.4) [0–99.4]**	**36.2 (9.8–65.0) [0–100]**	**0 (0–1.0) [0–97.7]**
Acute care		43	163.6 (79.0–210.6) [8.4–287.2]	54.6 (30.9–79.4) [0–98.5]	39.9 (15.1–63.1) [1.5–100]	0 (0–1.0) [0–97.7]

Clinic code, reason for visit	≥1 outdoor waiting area?	Individuals captured, n	Time captured per individual (minutes), median (IQR) [range]	Proportion of time spent indoors (%), median (IQR) [range]	Proportion of captured time spent outdoors (%), median (IQR) [range]	Proportion of captured time spent in unknown location (%), median (IQR) [range]
HIV/ART		56	128.0 (54.1–199.0) [7.4–325.1]	41.4 (31.5–70.6) [0–97.5]	55.2 (23.9–67.8) [0–100]	0 (0–0.5) [0–60.3]
Other chronic		7	161.6 (23.6–211.3) [16.3–225.4]	51.8 (20.9–70.7) [0–91.3]	48.2 (29.3–73.1) [8.7–100]	0 (0–0.4) [0–6.0]
TB		1	5.8 (5.8–5.8) [5.8–5.8]	0.3 (0.3–0.3) [0.3–0.3]	99.7 (99.7–99.7) [99.7–99.7]	0 (0–0) [0–0]
Accompanying a child		11	224.3 (40–268.0) [5.9–287.9]	74.6 (23.8–92.7) [0.4–93.2]	17.7 (6.8–45.2) [2.6–77.7]	0 (0–0.7) [0–97.0]
Accompanying an adult		11	64.8 (53.2–165.5) [13.8–320]	47.8 (0–77.3) [0–98.6]	26.1 (4.5–95.6) [1.4–100]	0 (0–4.4) [0–48.8]
Attending for someone else		5	65.2 (51.4–77.3) [7.7–234.1]	93.3 (52.3–93.5) [42.1–98.5]	5.7 (1.6–35.1) [1.5–57.4]	1.1 (0.5–4.9) [0–12.6]
Mother & child		33	202.5 (162.5–234.9) [58.9–352.9]	77.2 (62.3–94.1) [30.1–99.4]	14.3 (3.4–35.6) [0.4–69.9]	0.2 (0–1.3) [0–45.1]
**KZN4**	**Yes**	**262**	**106.7 (58.5–146.9) [5.6–333.1]**	**85.6 (50.4–100) [0–100]**	**9.4 (0–35.4) [0–99.2]**	**0 (0–0) [0–100]**
Acute care		24	56.6 (29.2–146.9) [5.6–227.6]	100 (76.6–100) [32.8–100]	0 (0–21.1) [0–67.2]	0 (0–0) [0–17.7]
HIV/ART		227	111.2 (61.2–147.0) [6.4–333.1]	83.7 (49.9–100) [0–100]	10.9 (0–35.8) [0–99.2]	0 (0–0) [0–100]
TB		5	62.8 (29.3–63.4) [28.6–142.3]	100 (100–100) [96.1–100]	0 (0–0) [0–3.9]	0 (0–0) [0–0]
Accompanying a child		2	130.6 (47.4–213.7) [47.4–213.7]	71.5 (43.1–100) [43.1–100]	28.5 (0–56.9) [0–56.9]	0 (0–0) [0–0]
Accompanying an adult		2	92.4 (38.9–145.8) [38.9–145.8]	67.4 (34.8–100) [34.8–100]	32.6 (0–65.2) [0–65.2]	0 (0–0) [0–0]
Mother & child		2	92.9 (47.4–138.4) [47.4–138.4]	72.0 (44.1–100) [44.1–100]	23.8 (0–47.7) [0–47.7]	4.1 (0–8.3) [0–8.3]
**KZN5**	**Yes**	**342**	**132.8 (58.4–203.5) [6.8–370.9]**	**86.2 (19.2–97.2) [0–100]**	**5.2 (1.7–40.8) [0–100]**	**0 (0–3.4) [0–99.7]**
Acute care		99	104.2 (58.1–185.1) [6.8–349.7]	89.0 (16.3–97.8) [0–99.8]	4.6 (1.3–40.8) [0–100]	0 (0–3.6) [0–99.7]
HIV/ART		112	156.3 (86.5–202.1) [10.8–370.9]	91.7 (64.9–97.0) [0–100]	4.6 (1.8–13.9) [0–100]	0 (0–3.5) [0–96.9]
Other chronic		11	55.0 (29.6–130.9) [9.3–336.0]	95.6 (81.5–96.9) [5.1–98.3]	4.4 (2.7–16.1) [1.7–94.9]	0 (0–0) [0–26.5]
TB		20	124.2 (74.4–162.5) [11.6–256.5]	46.0 (18.1–91.8) [0–99.2]	49.8 (8.2–81.9) [0.8–100]	0 (0–0) [0–8.3]
Accompanying a child		11	176.0 (90.5–226.1) [12.6–280.7]	31.5 (0–87.2) [0–99.6]	37.1 (10.9–100) [0.4–100]	0 (0–3.4) [0–61.6]
Accompanying an adult		18	89.2 (54.2–177.2) [16.0–214.8]	65.4 (4.8–98.4) [0.5–99.0]	2.4 (1.1–43.6) [0–96.9]	0 (0–37.0) [0–98.2]
Attending for someone else		10	163.0 (41.5–193.9) [13.3–310.3]	88.2 (32.3–96.2) [0–99.6]	6.8 (3.3–34.1) [0.4–100]	0 (0–0) [0–94.0]
Mother & child		61	150.1 (57.2–226.8) [7.1–321.7]	37.5 (1.7–97.8) [0–99.8]	7.2 (1.2–72.5) [0–100]	0 (0–24.3) [0–98.1]

Clinic code, reason for visit	≥1 outdoor waiting area?	Individuals captured, n	Time captured per individual (minutes), median (IQR) [range]	Proportion of time spent indoors (%), median (IQR) [range]	Proportion of captured time spent outdoors (%), median (IQR) [range]	Proportion of captured time spent in unknown location (%), median (IQR) [range]
**KZN6**	**No**	**209**	**85.5 (47.3–132.0) [5.8–323.1]**	**97.9 (93.8–99.0) [0–100]**	**1.8 (0.8–5.4) [0–100]**	**0 (0–0) [0–66.7]**
Acute care		86	66.3 (33.9–125.1) [5.8–323.1]	96.8 (93.1–98.8) [0–100]	1.9 (1.0–6.7) [0–100]	0 (0–0) [0–66.7]
HIV/ART		30	104.9 (35.1–156.9) [13.3–248.1]	98.5 (95.0–99.1) [34.0–100]	1.5 (0.7–4.9) [0–66.0]	0 (0–0) [0–4.1]
Other chronic		11	85.5 (25.2–113.6) [12.3–144.4]	97.5 (95.0–99.3) [87.4–99.6]	2.5 (0.7–5.0) [0.4–12.6]	0 (0–0) [0–0]
TB		7	118.2 (97.0–138.9) [63.9–163.8]	99.4 (98.4–100) [98.3–100]	0.6 (0–1.6) [0–1.7]	0 (0–0) [0–0]
Accompanying a child		15	113.7 (50.4–165.2) [11.5–261.1]	98.4 (95.7–99.3) [86.4–99.7]	1.6 (0.7–3.9) [0.3–13.6]	0 (0–0) [0–4.4]
Accompanying an adult		12	49.2 (19.1–88.0) [7.6–266.4]	83.1 (58.0–92.9) [17.3–100]	16.9 (7.1–42.0) [0–82.7]	0 (0–0) [0–4.6]
Attending for someone else		4	80.8 (47.7–105.2) [27.5–116.7]	98.3 (95.5–98.6) [92.8–98.8]	1.7 (1.4–4.5) [1.2–7.2]	0 (0–0) [0–0]
Mother & child		44	115.1 (61.1–165.9) [7.1–216.7]	98.1 (95.3–99.2) [61.0–100]	1.2 (0.6–3.1) [0–39.0]	0 (0–0) [0–27.1]
**WC1**	**No**	**307**	**145.9 (63.0–230.3) [6.0–381.6]**	**100 (99.7–100) [0–100]**	**0 (0–0) [0–100]**	**0 (0–0) [0–100]**
Acute care/HIV*		172	133.1 (62.7–215.8) [6.2–381.6]	100 (99.3–100) [0–100]	0 (0–0) [0–100]	0 (0–0) [0–100]
Other chronic		52	151.4 (58.8–214.3) [8.0–363.9]	100 (100–100) [0–100]	0 (0–0) [0–56.4]	0 (0–0) [0–97.7]
TB		7	213.8 (135.3–258.0) [74.1–325.7]	100 (100–100) [96.3–100]	0 (0–0) [0–3.7]	0 (0–0) [0–0]
Accompanying a child		18	203.4 (55.5–246.1) [33.7–306.6]	100 (100–100) [98.6–100]	0 (0–0) [0–1.4]	0 (0–0) [0–0.8]
Accompanying an adult		10	63.9 (38.3–190.3) [6.0–336.3]	100 (99.4–100) [93.6–100]	0 (0–0) [0–0.8]	0 (0–0) [0–6.4]
Attending for someone else		17	138.1 (31.1–221.0) [8.7–291.4]	100 (100–100) [91.1–100]	0 (0–0) [0–8.9]	0 (0–0) [0–0.6]
Mother & child		29	184.7 (106.9–242.7) [35.2–308.0]	100 (100–100) [93.5–100]	0 (0–0) [0–6.5]	0 (0–0) [0–2.6]
Not recorded		2	233.0 (144.4–321.5) [144.4–321.5]	99.1 (98.2–100) [98.2–100]	0.9 (0–1.8) [0–1.8]	0 (0–0) [0–0]
**WC2**	**No**	**64**	**124.4 (63.8–193.7) [14.0–363.6]**	**98.3 (88.0–99.5) [2.6–99.9]**	**0.8 (0.4–3.4) [0–37.6]**	**0 (0–1.1) [0–94.3]**
Acute care/HIV*		21	144.0 (89.1–208.5) [14.0–363.6]	98.7 (90.5–99.6) [2.6–99.9]	0.6 (0.4–3.0) [0.1–18.0]	0 (0–2.0) [0–94.3]
TB		3	118.8 (48.9–153.8) [48.9–153.8]	98.8 (97.6–99.6) [97.6–99.6]	1.2 (0.4–2.4) [0.4–2.4]	0 (0–0) [0–0]
Accompanying a child		12	74.6 (54.8–176.3) [52.2–264.6]	98.3 (81.9–99.3) [30.5–99.9]	1.7 (0.7–7.2) [0.1–27.1]	0 (0–0.3) [0–48.7]
Accompanying an adult		1	58.8 (58.8–58.8) [58.8–58.8]	81.1 (81.1–81.1) [81.1–81.1]	0.8 (0.8–0.8) [0.8–0.8]	18.1 (18.1–18.1) [18.1–18.1]

Clinic code, reason for visit	≥1 outdoor waiting area?	Individuals captured, n	Time captured per individual (minutes), median (IQR) [range]	Proportion of time spent indoors (%), median (IQR) [range]	Proportion of captured time spent outdoors (%), median (IQR) [range]	Proportion of captured time spent in unknown location (%), median (IQR) [range]
Mother & child		27	123.9 (66.3–193.8) [45.4–302.8]	98.1 (86.9–99.5) [43.3–99.9]	0.7 (0.3–6.7) [0–37.6]	0 (0–0.6) [0–56.6]
**WC3**	**No**	**111**	**154.6 (60.3–210.1) [5.8–323.5]**	**100 (99.2–100) [33.7–100]**	**0 (0–0.6) [0–56.4]**	**0 (0–0) [0–66.3]**
Acute care/HIV*		43	172.5 (117.1–200.8) [15.3–323.4]	100 (99.3–100) [33.7–100]	0 (0–0) [0–30.5]	0 (0–0) [0–66.3]
TB		15	32.2 (9.3–46.9) [5.8–250.1]	100 (99.1–100) [43.6–100]	0 (0–0) [0–56.4]	0 (0–0) [0–10.8]
Accompanying a child		22	197.7 (103.8–272.4) [31.6–323.5]	100 (98.1–100) [93.8–100]	0 (0–1.9) [0–6.2]	0 (0–0) [0–2.5]
Accompanying an adult		4	160.2 (69.2–212.6) [5.8–237.4]	83.0 (55.0–100) [44.0–100]	16.9 (0–44.9) [0–56.0]	0 (0–0.2) [0–0.3]
Attending for someone else		2	40.6 (34.0–47.1) [34.0–47.1]	91.3 (82.7–100) [82.7–100]	8.7 (0–17.3) [0–17.3]	0 (0–0) [0–0]
Mother & child		25	167.7 (80.4–201.4) [6.6–284.5]	100 (100–100) [86.1–100]	0 (0–0) [0–13.9]	0 (0–0) [0–0]
**WC6**	**No**	**137**	**71.1 (24.2–197.2) [5.7–361.1]**	**100 (100–100) [22.7–100]**	**0 (0–0) [0–77.3]**	**0 (0–0) [0–0]**
Acute care/HIV*		76	121.0 (37.3–246.5) [5.7–361.1]	100 (100–100) [22.7–100]	0 (0–0) [0–77.3]	0 (0–0) [0–0]
Other chronic		10	97.0 (44.5–168.0) [14.8–239.0]	100 (100–100) [100–100]	0 (0–0) [0–0]	0 (0–0) [0–0]
TB		12	23.7 (13.9–50.4) [9.0–225.6]	100 (100–100) [99.9–100]	0 (0–0) [0–0.1]	0 (0–0) [0–0]
Accompanying a child		13	168.9 (71.9–194.8) [9.6–323.9]	100 (100–100) [97.1–100]	0 (0–0) [0–2.9]	0 (0–0) [0–0]
Accompanying an adult		11	58.1 (20.2–112.3) [14.5–137.4]	100 (100–100) [92.6–100]	0 (0–0) [0–7.4]	0 (0–0) [0–0]
Attending for someone else		9	32.1 (22.8–58.3) [6.3–61.5]	100 (97.3–100) [82.8–100]	0 (0–2.7) [0–17.2]	0 (0–0) [0–0]
Mother & child		6	81.3 (20.6–92.9) [17.0–112.0]	100 (100–100) [97.6–100]	0 (0–0) [0–2.4]	0 (0–0) [0–0]

*Acute care and HIV combined for Western Cape clinics due to error in data collection (see Methods in main article for details)

ART: antiretroviral therapy; IQR: interquartile range; KZN: KwaZulu-Natal; TB: tuberculosis; WC: Western Cape

3.4. Occupancy density

Table I. Occupancy density by room area and room volume in three indoor spaces in each of clinics KZN2, KZN6, and WC1 from 0800–1345*

Clinic	Space	Designation	Area, m^2	Mean height, m†	Maximum height, m	Volume, m^3	Occupancy (n), median (IQR) [range]	Occupancy density by room area (persons/m^2), median (IQR) [range]	Occupancy density by room volume (persons /m^3), median (IQR) [range]	Comments
**KZN6**	A	Pre-registration/pre-vitals waiting area	65.6	2.5	2.5	162.8	41 (35–45) [23–57]	0.63 (0.53–0.69) [0.35–0.87]	0.25 (0.21–0.28) [0.14–0.35]	All spaces have relatively low, flat ceilings
	B	Pre-consultation waiting area	19.4	2.5	2.5	48.2	19 (11–27) [0–32]	0.98 (0.57–1.39) [0–1.65]	0.39 (0.23–0.56) [0–0.66]	
	C	Pre-consultation waiting area	15.9	2.5	2.5	25.6	26 (21–28) [11–34]	1.63 (1.32–1.76) [0.69–2.14]	1.02 (0.82–1.09) [0.43–1.33]	
**KZN2**	D	Pre-registration waiting area	49.7	2.7	2.7	136.4	30 (19–34) [7–51]	0.60 (0.38–0.68) [0.14–1.03]	0.22 (0.14–0.25) [0.05–0.37]	All spaces have relatively low, flat ceilings
	E	Corridor used as pre-consultation waiting area	20.4	2.6	2.6	42.7	11 (9–13) [0–19]	0.54 (0.44–0.64) [0–0.93]	0.21 (0.17–0.24) [0–0.36]	
	F	Combined pre-vitals waiting area, vitals administration area, and registration area	16.3	2.6	2.6	54.4	14 (9–19) [0–29]	0.86 (0.55–1.16) [0–1.78]	0.33 (0.21–0.45) [0–0.68]	
**WC1**	G	Pre-registration waiting area	129.5	3.5	4.2	368.2	52 (45–56) [29–71]	0.40 (0.35–0.43) [0.22–0.55]	0.13 (0.10–0.15) [0–0.19]	All spaces have relatively high, sloping ceilings
	H	Pre-vitals waiting area	53.2	5.3	5.9	272.8	29 (21–34) [5–43]	0.55 (0.39–0.64) [0.09–0.81]	0.10 (0.07–0.12) [0–0.16]	
	I	Pre-consultation waiting area	37.7	4.2	5.9	169.1	23 (18–25) [8–34]	0.61 (0.48–0.66) [0.21–0.90]	0.14 (0.09–0.15) [0–0.20]	

*Data available only from 0830 for clinic KZN6
†Some spaces had sloping ceilings and/or areas with lower flat ceilings
IQR: interquartile range

Appendix 4. Additional discussion

Table J. Summary of previous studies estimating waiting times in South African PHC clinics

First author, year published	Location	Number of facilities	Facility type	Dates of data collection	Data collection methods	Key findings
Stime, 2018[17]	Durban, KZN	1	HIV/TB/STI outpatient centre	2016	Time and motion (direct observation)	Median time in clinic 01:48 (range 00:28–04:35) for STI care (n = 39) and 02:46 (range 01:25–03:50) for those attending for HIV care (n = 27).<br>HIV 'fast track' slightly reduced time (median 02:24 [range 00:14–04:43; n = 28]) and use of point-of-care diagnostics for STIs (instead of syndromic management) increased time spent (median 04:02; n = 9).
Egbujie, 2018[18]	KZN	9	PHC clinics	2014–15	Adapted NDoH waiting time approach	Implemented a range of Ideal Clinic recommendations.<br>Pre-intervention median time in clinic 01:56 (IQR 01:06–02:48); n = 860<br>Post-intervention median time in clinic 02:02 (IQR 01:21–03:24); n = 903<br>Multivariable analysis: longer waiting times associated with earlier arrival time and higher patient/nurse ratio.
Daniels, 2017[19]	WC	22	PHC clinics	2007, 2011	Standard WTSE approach	2007 survey: median waiting time* 01:16 (IQR 00:37–02:05); median 233 patients attending per day per clinic<br>2011 survey: median waiting time* 00:55 (IQR 00:28–01:54); median 255 patients attending per day per clinic
Bachmann, 1997[1]	Khayelitsha, WC	1	CHC	1993	Paper-based waiting time approach	Median time in clinic: 2.6 (IQR 1.7–3.8) hours and 4.1 (IQR 2.9–4.9) hours for those attending for 'preventive' (n = 368) and 'curative' (n = 416) care, respectively.

*Only time spent waiting, does not include time spent receiving services

CHC: community health centre; IQR: interquartile range; KZN: KwaZulu-Natal; NDoH: National Department of Health; PHC: primary health care; STI: sexually transmitted infection; TB: tuberculosis; WC: Western Cape; WTSE: Waiting Time and Systems Efficiency

Appendix 5. Acknowledgments

Table K. The extended *Umoya omuhle* team, institutions, and roles (listed alphabetically by surname)

Name	Institution/s	Role
Siphokazi Adonisi	UCT	Research Assistant
Kathy Baisley	LSHTM; AHRI	Co-investigator
Peter Beckwith	LSHTM; UCT	Research fellow
Fiammetta Bozzani	LSHTM	Co-investigator
Amy Burdzik	UCT	Occupational health
Adrienne Burrough	LSHTM	Project Manager
Nkosingiphile Buthelezi	AHRI	Research Assistant
Xolile Buthelezi	AHRI	Diagnostic Lab Manager
Ruvimbo Chigwanda	UCT	Administration
Christopher Colvin	UCT	Co-investigator
PIP CRAs	AHRI	Clinic research Assistants
Njabulo Dayi	AHRI	Research Data Manager
Arminder Deol	LSHTM	Mathematical modeller
Karina Diaconu	QMU	Co-investigator
Siphephelo Dlamini	AHRI	Nursing Manager
Yutu Dlamini	AHRI	Research Assistant
Raveshni Durgiah	AHRI	Grants office
Anita Edwards	AHRI	Head: Scientific Support
Jennifer Falconer	QMU	Research Assistant
Kitty Flynn	QMU	Administrator
Patrick Gabela	AHRI	Clinical Research Data Coordinator
Dickman Gareta	AHRI	Head: Research Data Management
Awethu Gawulekapa	UCT	Research Assistant
Harriet Gliddon	AHRI; UCL	Research Assistant
Bavashni Govender	UKZN	Administration
Indira Govender	LSHTM; AHRI	Co-investigator
Alison Grant	LSHTM; AHRI	Principal investigator
Meghann Gregg	LSE	Research Fellow
Emmerencia Gumede	AHRI	Research Assistant
Sashin Harilall	AHRI	Grants office
Kobus Herbst	AHRI	Chief Information Officer
Tamia Jansen	UCT	Research Assistant
Seonaid Kabiah	UCT	Research Assistant
Idriss Kallon	UCT	Post-doctoral researcher
Aaron Karat	LSHTM	Co-investigator
Hannah Keal	AHRI	Communications
Suzanne Key	UCT	Occupational health
Zama Khanyile	UKZN	Research Assistant
Mandla Khoza	AHRI	Clinic Research Assistant
Nozi Khumalo	AHRI	Systems Engineer
Zilethile Khumalo	AHRI	Research Assistant
Karina Kielmann	QMU	Co-principal investigator

Name	Institution/s	Role
Nondumiso Kumalo	AHRI	Clinic Research Assistant
Richard Lessells	AHRI	Epidemiologist
Nokuthula Lushaba (deceased)	UKZN	Administration
Sithembiso Luthuli	AHRI	Research Assistant
Sinethemba Mabuyakhulu	AHRI	Clinic Research Assistant
Hayley MacGregor	IDS	Co-investigator
Nonhlanhla Madlopha	AHRI	Research Assistant
Aphiwe Makalima	UCT	Administration
Tacha Malaza	AHRI	PIP CRA
Sifundesihle Malembe	AHRI	Research Assistant
Godfrey Manuel	UCT	Transport
Nonhlanhla Maphumulo	UKZN	Administration
Precious Mathenjwa	UCT	Research Assistant
Sanele Mbuyazi	AHRI	PIP CRA
Nicky McCreesh	LSHTM	Co-investigator
Claire McLellan	QMU	Administrator
Simphiwe Mdluli	AHRI	PIP CRA
Thabile Mkhize	AHRI	Transport
Duduzile Mkhwanazi	AHRI	Research Assistant
Zinhle Mkhwanazi	AHRI	Research Assistant
Zodwa Mkhwanazi	AHRI	Research Assistant
Anathi Mngxekeza	UCT	Research Assistant
Tshwaraganang Modise	AHRI	Research Data
Sashen Moodley	AHRI	Microbiology Laboratory Supervisor
Samantha Moyo	UCT	Research Assistant
Silindile Mthembu	AHRI	Clinic Research Assistant
Nozipho Mthethwa	AHRI	Research Assistant
Siphesihle Mthethwa	AHRI	Procurement Coordinator
Sphiwe Mthethwa	AHRI	Research Assistant
Sanele Mthiyane	AHRI	Research Assistant
Vanisha Munsamy	AHRI	Grants office
Sinead Murphy	UCT	Research Assistant
Thomas Murray	AHRI	Research assistant
Senzile Myeni	AHRI	PIP CRA
Tevania Naidoo	AHRI	Procurement
Nompilo Ndlela	AHRI	Research Assistant
Zama Ndlela	AHRI	PIP CRA
Thandekile Nene	AHRI	Research Assistant
Phumla Ngcobo	AHRI	Communications
Nzuzo Ntombela	AHRI	Research Data Systems Service Manager
Sabelo Ntuli	AHRI	GIS Coordinator
Nompumulelo Nyawo	AHRI	Human resources
Phumzile Nywagi	UCT	Research Assistant
Stephen Olivier	AHRI	Statistician

Name	Institution/s	Role
Justin Parkhurst	LSE	Co-investigator
Alex Pym	AHRI	Co-investigator
Yolanda Qeja	UCT	Research Assistant
Anand Ramnanan (deceased)	AHRI	Procurement
Sharmila Rugbeer	UKZN	Administration
Janet Seeley	LSHTM	Co-investigator
Aruna Sevakram	AHRI	Scientific support
Sizwe Sikhakane	AHRI	Transport
Zizile Sikhosana	AHRI	Somkhele Laboratory Supervisor
Theresa Smit	AHRI	Head: Diagnostic Research
Thandeka Smith	UKZN	Research Assistant
Naomi Stewart	LSHTM	Communications
Alison Swartz	UCT	Co-investigator
Amy Thomas	LSHTM	Communications
Siphosethu Titise	UCT	Research Assistant
Anna Vassall	LSHTM	Co-investigator
Marlise Venter	AHRI	Facilities Administrator
Anna Voce	UKZN	Co-investigator
Richard White	LSHTM	Co-investigator
Tom Yates	Imperial	Co-investigator
Precious Zulu	AHRI	Administration
Gimenne Zwama	QMU	Research Fellow

AHRI: Africa Health Research Institute; IDS: Institute of Development Studies; LSE: London School of Economics and Political Science; LSHTM: London School of Hygiene & Tropical Medicine; QMU: Queen Margaret University; UCT: University of Cape Town; UKZN: University of KwaZulu-Natal;

Appendix 6. References

1 Bachmann MO, Barron P. Why wait so long for child care? An analysis of waits, queues and work in a South African urban health centre. *Trop Doct*. 1997;**27**(1):34–8.

2 South Africa National Department of Health. Ideal Clinic Integrated Clinical Services Management Manual. https://www.idealhealthfacility.org.za/docs/Integrated%20Clinical%20Services%20Management%20%20Manual%205th%20June%20FINAL.pdf (accessed 2010 Jul 2)

3 Reagon G, Igumbor E. Strengthening Health Systems through training of Health Care Providers in the conduct of Routine Waiting Time and System Efficiency Surveys. *Stud Health Technol Inform*. 2010;590–4.

4 Singman EL, Haberman CV, Appelbaum J, et al. Electronic Tracking of Patients in an Outpatient Ophthalmology Clinic to Improve Efficient Flow: A Feasibility Analysis and Benchmarking Study. *Qual Manag Health Care*. 2015;**24**(4):190–9.

5 Montanari A, Tian Z, Francu E, et al. Measuring Interaction Proxemics with Wearable Light Tags. *Proc ACM Interact Mob Wearable Ubiquitous Technol*. 2018;**2**(1):1–30.

6 Aharony N, Pan W, Ip C, Khayal I, Pentland A. Social fMRI: Investigating and shaping social mechanisms in the real world. *Pervasive Mob Comput*. 2011;**7**(6):643–59.

7 Aiello AE, Simanek AM, Eisenberg MC, et al. Design and methods of a social network isolation study for reducing respiratory infection transmission: The eX-FLU cluster randomized trial. *Epidemics*. 2016;**15**:38–55.

8 Rodriguez M, Sivic J, Laptev I, Audibert J-Y. Data-driven crowd analysis in videos. In: *2011 International Conference on Computer Vision*. 2011. p. 1235–42.

9 Rodriguez M, Laptev I, Sivic J, Audibert J-Y. Density-aware person detection and tracking in crowds. In: *2011 International Conference on Computer Vision*. 2011. p. 2423–30.

10 Rodriguez M, Ali S, Kanade T. Tracking in unstructured crowded scenes. In: *2009 IEEE 12th International Conference on Computer Vision*. 2009. p. 1389–96.

11 Committee on Traffic Flow Theory and Characteristics, Federal Highway Administration. Traffic Flow Theory. 1975. http://tft.eng.usf.edu/docs.htm (accessed 2021 Apr 12)

12 Armony M, Israelit S, Mandelbaum A, Marmor YN, Tseytlin Y, Yom-Tov GB. On Patient Flow in Hospitals: A Data-Based Queueing-Science Perspective. An Extended Version. *Stoch Syst*. 2015;**5**(1):146–94.

13 Carmen R sa, Van Nieuwenhuyse I. Improving Patient Flow in Emergency Departments with OR Techniques: A Literature Overview. *SSRN Electron J*. 2014;

14 Higginson I. Emergency department crowding. *Emerg Med J*. 2012;**29**(6):437–43.

15 Bean DM, Taylor P, Dobson RJB. A patient flow simulator for healthcare management education. *BMJ Simul Technol Enhanc Learn*. 2019;**5**(1):46–8.

16 McAdams D. Changing the Game of Patient Experience: Insights from Game Theory. 2014. https://faculty.fuqua.duke.edu/~dm121/press/HSAC%20Patient%20Experience%20Final.pdf (accessed 2021 Apr 12)

17 Stime KJ, Garrett N, Sookrajh Y, et al. Clinic flow for STI, HIV, and TB patients in an urban infectious disease clinic offering point-of-care testing services in Durban, South Africa. *BMC Health Serv Res*. 2018;**18**(1):363.

18 Egbujie BA, Grimwood A, Mothibi-Wabafor EC, et al. Impact of 'Ideal Clinic' implementation on patient waiting time in primary healthcare clinics in KwaZulu-Natal Province, South Africa: A before-and-after evaluation. *S Afr Med J*. 2018;**108**(4):311.

19 Daniels J, Zweigenthal V, Reagon G. Assessing the impact of a waiting time survey on reducing waiting times in urban primary care clinicss in Cape Town, South Africa. *J Public Health Afr*. 2017;**8**:23–9.
