# Supplementary material for: Estimating waiting times, patient flow, and waiting room occupancy density as part of tuberculosis infection prevention and control research in South African primary health care clinics
Source: PLOS Glob Public Health. 2022 Jul 20;2(7):e0000684. doi: 10.1371/journal.pgph.0000684 (PMC10021248; doi:10.1371/journal.pgph.0000684)
Supplement: Attachment — Submitted filename: PGPH-D-21-00590_ResponseToReviewers_20220528.pdf [file pgph.0000684.s002.pdf]

Dr Aaron Karat
TB Centre, London School of Hygiene & Tropical Medicine
Keppel Street, London WC1E 7HT
aaron.karat@lshtm.ac.uk

Dr Reuben Kiggundu,

Academic Editor,

PLoS Global Public Health

28 May 2022

Dear Dr Kiggundu,

Re: PGPH-D-21-00590
Waiting times, patient flow, and occupancy density in South African primary health care clinics: implications for infection prevention and control; Karat AS, et al.

Thank you for the opportunity to respond to the thoughtful reviews of this article, all of which have helped strengthen it. Please convey our thanks to the reviewers for their time.

Editor and reviewer comments are reproduced overleaf in shaded text, followed by our responses, unshaded. All page and line numbers cited below correspond to the file named "Manuscript_R1(clean).docx" unless otherwise specified. Original text is framed in grey, dashed lines, and revised text in black, solid lines.

Editor

Comment 1

In your ethics statement in the manuscript , please state whether the IRB or ethics committee waived the requirement for informed consent.

Response: Thank you, this has been amended as shown below.

Editor, comment 1, amendment 1 of 1 (Ethics approval)

Original text

"Identifiable data were not collected from participating individuals and written informed consent was therefore not requested. This study received ethical approval from…"

Amended text (page 21, line 457–459)

"Identifiable data were not collected from participating individuals and written informed consent was therefore not requested. This study received ethical approval (including waivers for written informed consent) from…""

Comment 2

Please include additional information regarding the survey or questionnaire used in the study and ensure that you have provided sufficient details that others could replicate the analyses. For instance, if you developed a questionnaire as part of this study and it is not under a copyright more restrictive than CC-BY, please include a copy, in both the original language and English, as Supporting Information.

Response: Data collection tools for attendees and staff are presented in full in Supplementary figure 2 (page 9 of supplementary material).

Comment 3

Please amend your detailed Financial Disclosure statement. This is published with the article, therefore should be completed in full sentences and contain the exact wording you wish to be published.

State what role the funders took in the study. If the funders had no role in your study, please state: "The funders had no role in study design, data collection and analysis, decision to publish, or preparation of the manuscript."

Response: This statement has been added to the text under the 'Funding' subheading (lines 477–478).

Comment 4

Please update your Competing Interests statement. If you have no competing interests to declare, please state: "The authors have declared that no competing interests exist."

Response: This has been updated as shown below.

Editor, comment 4, amendment 1 of 1 (Competing interests)

Original text

"The authors declare that they have no competing interests. All authors have completed the ICMJE uniform disclosure form at www.icmje.org and declare: financial support from the Economic and Social Research Council (UK) and from The Bloomsbury SET (Research England) for the submitted work; no financial relationships with any organizations that might have an interest in the submitted work in the previous three years; and no other relationships or activities that could appear to have influenced the submitted work."

Amended text (page 22, line 470)

"The authors have declared that no competing interests exist."

Comment 5

In the online submission form, you indicated that your data will be submitted to a repository upon acceptance. We strongly recommend all authors deposit their data before acceptance, as the process can be lengthy and hold up publication timelines. Please note that, though access restrictions are acceptable now, your entire data will need to be made freely accessible if your manuscript is accepted for publication.

This policy applies to all data except where public deposition would breach compliance with the protocol approved by your research ethics board. If you are unable to adhere to our open data policy, please kindly revise your statement to explain your reasoning and we will seek the editor's input on an exemption. Please be assured that, once you have provided your new statement, the assessment of your exemption will not hold up the peer review process.

Response: Data have been uploaded to the ReShare repository and should be accessible by the end of June 2022. We have amended the text as show below.

Editor, comment 5, amendment 1 of 1 (Availability of data and materials)

Original text

"Data will be available from LSHTM DataCompass. Questionnaires used are provided in the supplementary material."

Amended text (page 21, line 467–468)

"Data are available from the ReShare repository (http://doi.org/10.5255/UKDA-SN-854435). Questionnaires used are provided in the supplementary material."

Comment 6

We have noticed that you have uploaded supporting information but you have not included a list of legends. Please add a full list of legends for all supporting information files (including figures, table and data files) after the references list.

Response: Thank you, these have been added (lines 667–701).

Comment 7

The link between the study data (patient waiting time, etc.) is not clearly linked to the IPC.

The data collection and methods sections do not describe if any data about IPC outcomes/indicators was collected. How then do the authors link their findings to IPC? It seems IPC was not part of the study and the authors are making inferences in regards to IPC based on the findings of patient flow in the clinic. If this is the case, I suggest we add a statement in the discussion sections so we have this clarity.

Revise the recommendation section to show the connection between the study topic and IPC.

Currently, the title of the paper is not reflected in the discussion section and the recommendations. If this revision is not possible, please consider revising the title of the manuscript.

Additionally, if we maintain IPC in the title, the authors should add the word TB, so that it can read as ''TB IPC''.

The authors should discuss the implications of their findings to IPC. For example, they state that ''early arrival, being female, and attending with a young child lead to longer wait times? However, it would be important to discuss the implications of these variables on the IPC.

Response: Thank you, we have addressed the above comments together, along with Reviewer 2's major comment #1. We recognise that the title of the paper did not match the contents of the discussion/recommendations, specifically around IPC interventions. As you are aware, patient flow is only one of many factors that affect the risk of respiratory pathogen transmission. The data presented in this article were collected as part of the *Umoya omuhle* study (a multidisciplinary examination of TB IPC implementation) and were used to develop mathematical models to estimate the effectiveness of seven TB IPC interventions (results reported in McCreesh et al., *BMJ Glob H* 2021 [PMID 34697087]).

Given the continuing high incidence of TB in health care workers in high burden countries and the decentralisation of DR-TB care from hospitals to primary health care clinics, one of the aims of the *Umoya omuhle* study was to find shortcomings in current IPC policy and implementation (see Kielmann et al., *BMC Infect Dis Pov* 2020 [PMID 32450916]). One of the gaps we identified was the lack of attention given to 'patient flow' as part of IPC practice or research, despite recommendations in 2009 World Health Organization TB IPC guidelines to "*rethink the use of available spaces*" (page 12) and to focus on "*minimizing time spent in health facilities*" (page 13; https://apps.who.int/iris/bitstream/handle/10665/44148/9789241598323_eng.pdf;sequence=1). The system dynamics modelling approach taken by the *Umoya omuhle* study (Diaconu et al. [manuscript submitted], summary provided in this research brief) also meant that we focused on 'higher order' phenomena that may drive transmission but are not conventionally thought of as part of IPC. One of the critical factors identified by workshop participants was bottlenecks in patient flow, which were thought likely to be a key driver of transmission of *Mtb* and other respiratory pathogens in clinics.

As such, we consider that the issues discussed in this paper are extremely relevant to the implementation of IPC for *Mtb* and other respiratory pathogens. However, given the wide variety of factors that affect risk of transmission, we do not think that specific IPC recommendations can be made solely on the basis of these data. Because the relevance to IPC of overcrowding at health facilities has been recognised since at least 2009, we do not consider it necessary to explain this in detail in the manuscript, but have nonetheless made a number of changes to the abstract, introduction, and discussion to better reflect this link (please see tracked changes manuscript for details of minor changes).

As suggested, we have also revised the title of the article to read "*Estimating waiting times, patient flow, and waiting room occupancy density as part of tuberculosis infection prevention and control research in South African primary health care clinics*". We believe that this framing as a hypothesis-generating exercise and attempt to evaluate measurement approaches (as reflected in the study's aims [original manuscript lines 62–66]) is more accurate and will better guide readers as to what to expect.

Editor, comment 7, amendment 1 of 3 (Title)

Original title

"Waiting times, patient flow, and occupancy density in South African primary health care clinics: implications for infection prevention and control"

Amended title

"Estimating waiting times, patient flow, and waiting room occupancy density as part of tuberculosis infection prevention and control research in South African primary health care clinics"

Editor, comment 7, amendment 2 of 3 (Abstract)

Original text

"Transmission of respiratory pathogens, such as *Mycobacterium tuberculosis* and severe acute respiratory syndrome coronavirus 2, is more likely during close, prolonged contact and when sharing a poorly ventilated space. In clinics in KwaZulu-Natal (KZN) and Western Cape (WC), South Africa, we estimated clinic visit duration, time spent indoors and outdoors, and occupancy density of waiting rooms."

Amended text (page 2, lines 5–11)

"Transmission of respiratory pathogens, such as Mycobacterium tuberculosis and severe acute respiratory syndrome coronavirus 2, is more likely during close, prolonged contact and when sharing a poorly ventilated space. Reducing overcrowding of health facilities is a recognised infection prevention and control (IPC) strategy; reliable estimates of waiting times and 'patient flow' would help guide implementation. As part of the Umoya omuhle study, we aimed to estimate clinic visit duration, time spent indoors versus outdoors, and occupancy density of waiting rooms in clinics in KwaZulu-Natal (KZN) and Western Cape (WC), South Africa."

Editor, comment 7, amendment 3 of 3 (Abstract)

Original text

"Longer waiting times were associated with early arrival, being female, and attending with a young child. Attendees generally waited where they were asked to. Regular estimation of occupancy density (as proxy for patient flow) may help staff assess for risk of infection transmission and guide intervention to reduce time spent in risky spaces."

Amended text (page 2, lines 25–27)

"In this study, longer waiting times were associated with early arrival, being female, and attending with a young child. Occupancy of waiting rooms varied substantially between rooms and over the clinic day. Regular, light-touch estimation of occupancy density may help guide interventions to improve patient flow."

Reviewer #1

Comment 1

It would be useful to describe the characteristics of the primary health care clinic level of care in further detail. In the supplementary materials the authors also show that there are both community health centres (CHC) and primary health centres (PHC) in their data set. The variable characteristics which make the two types of sites distinct (if they exist) should be discussed.

Response: Thank you, we have added text to the introduction and results as shown below.

Reviewer 1, comment 1, amendment 1 of 2 (Introduction)

Original text

"Estimating time spent in South African PHC clinics

The nearly 3,500 PHC clinics …"

Amended text (page 3, line 46–54)

"Estimating time spent in South African PHC clinics

PHC clinics in South Africa are the main interface between the public health system and the population. Most services at PHC level are nurse-led and include the majority of routine care for pregnant women, children (including immunisations), and people living with HIV, drug-sensitive TB, and non-communicable diseases. PHC clinics also provide a range of acute services (from minor injuries to infections) and voluntary testing for HIV and TB. Some larger facilities (offering a 24-hour maternity service, casualty, and a short stay ward) are known as community health centres (CHCs), though these services are also offered by some PHC clinics. In this article, the term 'PHC clinic' is used to include CHCs unless otherwise specified.

The nearly 3,500 PHC clinics…"

Reviewer 1, comment 1, amendment 2 of 2 (Results)

Original text

"Twelve datasets were available for analysis from 11 clinics: six in KZN and five in WC (Table 1; clinic WC4 could not be visited for logistical reasons and clinic KZN1 was visited for a second time to attempt better coverage). Data were collected…"

Amended text (page 10, line 200)

"Twelve datasets were available for analysis from 11 clinics: six in KZN and five in WC (Table 1; clinic WC4 could not be visited for logistical reasons and clinic KZN1 was visited for a second time to attempt better coverage). Eight facilities were PHC clinics and three were CHCs (Supplementary table 5). Data were collected…"

Comment 2

It is unclear why the number of clinic attendees (2,903) different than the total number of attendees captured in the analysis for proportion of time spent indoors vs outdoors (2,190)? If it is because certain sites were excluded from the review due to characteristic the authors should clearly state this in their methods. For example, if this is due to Individuals with a total captured visit time of less than five minutes were excluded, as they were considered likely to have discarded their barcode. (LINE 151) then it seems reasonable those individuals should also be excluded from the overall analysis of time spent in clinic analysis as well?

Response: Thank you for this comment. The key difference between the two analyses is the use of 'original' vs. imputed data. We used 'original' data (without imputation) to describe the time spent indoors vs. outdoors (n = 2,190) and hence excluded individuals with very short visits who may have discarded their barcodes. A more accurate description would have been "individuals with a total captured time of less than five minutes and no record of having left the clinic" (this has now been changed in the manuscript – see below). When conducting the analysis for time spent in clinic, however, we used multiply imputed data, and all individuals with an arrival time but no recorded departure time were assigned a range of possible departure times as part of the imputation process.

We would also refer the reviewer to Supplementary table 3 (page 10 of the supplementary material) where we provide details of which clinics were included/excluded from each analysis, as well as any additional exclusion criteria applied.

We hope that this addresses the reviewer's concerns, and recognise that this could have been better explained in the manuscript. We have made minor edits to the text for clarity, as shown below.

Reviewer 1, comment 2, amendment 1 (Methods)

Original text

"Proportion of time spent indoors vs. outdoors

Non-imputed data were used from clinics where a scanner had been positioned at all facility entrance/s and all indoor/outdoor doorways. Individuals with a total captured visit time of less than five minutes were excluded, as they were considered likely to have discarded their barcode."

Amended text (page 8, lines 159–163)

"Proportion of time spent indoors vs. outdoors

Non-imputed data were used. Data were excluded 1) from clinics where a scanner had not been positioned at all facility entrances and all indoor/outdoor doorways and 2) from individuals with a total captured visit time of less than five minutes and no record of having left the clinic, as they were considered likely to have discarded their barcode (Supplementary table 3)."

Comment 3

There is a very significant number of missing data points related both to time of arrival (40%) and time of departure (35%). Although I understand the authors explain the use of multiple imputation to try and resolve this issue, the authorship team should spend some further time discussing the serious limitation of having such a significant amount of missing data and the impact that has on their given results. On line 240 it is also not clear how there are unknown locations included in the data set, further details would be useful here.

Response: Thank you. The designation of 'unknown' locations is described in the methods (original manuscript lines 151–154: *"…for each barcode scan recorded, the individual's location in the time preceding the scan was categorised as 'indoors', 'outdoors', or 'unknown' (if they appeared to have moved between two unconnected locations, indicating a missing barcode scan) based on the location of their previous scan…"*). For example, if an individual was scanned at one indoor location and next at another (unconnected) indoor location without being recorded as having passed through either a connecting door or an internal/external door, we were unable to assess if they had spent the intervening period in an indoor or outdoor location, and therefore categorised their location during this period as 'unknown'. We have added a line in the results to refer readers back to the description in the methods (see below).

We acknowledge the reviewer's point about the significance of the missing data (also raised by Reviewer 2). This was discussed to some extent in the 'limitations' section of the manuscript (original manuscript lines 405–412), but we have now expanded this description, as shown below.

Reviewer 1, comment 3, amendment 1 of 2 (Results)

Original text

"The 2,190 clinic attendees included in this analysis (≥5 minutes captured; 10 visits; 9 clinics) spent a median 95.6% (IQR 45.6–100) of their time indoors and a median 2.5% (IQR 0–35.3) outdoors (Supplementary table 7), with the remainder in unknown locations."

Amended text (page 13, line 251–255)

"The 2,190 clinic attendees included in this analysis (≥5 minutes captured; 10 visits; 9 clinics) spent a median 95.6% (IQR 45.6–100) of their time indoors and a median 2.5% (IQR 0–35.3) outdoors (Supplementary table 7), with the remainder in unknown locations (locations were classed as 'unknown' when it could not be assessed if the individual was indoors or outdoors – see Methods)."

Reviewer 1, comment 3, amendment 2 of 2 (Strengths and limitations)

Original text

"Starting data collection after some individuals had arrived and stopping data collection at 1400 (because of logistical restrictions) reduced the numbers of individuals whose data could be used to estimate total waiting time, requiring the use of multiple imputation to deal with missing data. Multiple imputation

assumes that the data are missing at random, which means that the observed values can be used to predict the missing values. However, if the assumption is incorrect, the results may be biased. Furthermore, the validity of results derived from multiply imputed data depend on the appropriateness of the imputation model. Future exercises should, at a minimum, continue to record clinic exits for as long as possible. Because of variability between and within clinics, and because data were collected on only one day from almost all clinics, estimates presented here should not be considered representative of the two provinces, types of clinics, or the clinics themselves."

Amended text (page 20, line 423–426)

"Starting data collection after some individuals had arrived and stopping data collection at 1400 (because of logistical restrictions) reduced the numbers of individuals whose data could be used to estimate total waiting time, requiring the use of multiple imputation to deal with missing data and reduce the risk of bias. Multiple imputation assumes that the data are missing at random, which means that the observed values can be used to predict the missing values. However, if the assumption is incorrect, the results may be biased. Furthermore, the validity of results derived from multiply imputed data depend on the appropriateness of the imputation model. Future similar exercises should, at minimum, continue to record clinic exits for as long as possible. Because of 1) the amount of missing data, 2) the variability between and within clinics, and 3) because data were collected on only one day from almost all clinics (seasonal changes in symptom/disease prevalence are also likely to affect rates of clinic attendance and therefore patient flow and waiting times), reported estimates should not be considered representative of the two provinces, types of clinics, or the clinics themselves."

Comment 4

It is not clear if the authors considered the impact of their data collection cut off time (at 2pm each day) on their findings. How do they account for the fact that patients who arrive at 12pm or 1pm will only have one hour of data collection time before the end of the day? The results indicating that attendees who arrived early in the morning spent longer in the clinic might just mean that their full visit was captured during the data collection period, whilst those who arrived later in the day only had a portion of their wait times captured. As one of the primary claims of this study (early arrival is associated with longer wait times) this seems like a critical issue which should be adequately explained.

Response: We acknowledge that this is a major limitation of the study (as discussed in the limitations [original manuscript lines 405–412]), but because the waiting times analysis was conducted using imputed data (original manuscript lines 133–135), we contend that this helps reduce the effects of this methodological limitation. Details of the imputation are provided in section 2.2.1 of the supplementary material. A key point is that this analysis used only two time stamps for each individual: time of arrival and time of departure. Therefore, individuals who arrived later (e.g., at 12pm) and who were still in the clinic at

the end of data collection (2pm) were assigned a range of possible departure times as part of the imputation. The range of visit durations derived from the imputed data were then included in the regression model. In a similar way, individuals who arrived before the start of data collection had a range of possible arrival times generated through imputation, and the derived range of visit durations were included in the model.

We hope that this addresses the reviewer's concerns. We have added to the limitations (see above) and made small changes to the methods, as shown below.

Reviewer 1, comment 4, amendment 1 (Methods)

Original text

"Time spent in clinic

A large number of data were missing for arrival and departure times because several individuals were already in the clinic when data collection began or remained in the clinic when data collection ended and therefore did not have their arrival and/or departure time recorded. Multiple imputation was used to generate arrival and/or departure times for individuals in whom one or both was not recorded (see Appendix 2.2.1 for details).

Data were excluded from this analysis where clinic entry and exit were not recorded consistently (because the research team was too small to monitor all entrances and exits of the clinic). Using multiply-imputed data (n = 20 imputations),"

Amended text (page 7, lines 141–149)

"Time spent in clinic

A large number of data were missing for arrival and departure times because some individuals were either 1) already in the clinic when data collection began or 2) remained in the clinic when data collection ended. These individuals therefore did not have their arrival and/or departure time recorded. Multiple imputation was used to generate arrival and/or departure times for individuals in whom one or both was not recorded (see Appendix 2.2.1 for details).

Data were excluded from this analysis from clinics where clinic entry and exit were not recorded consistently (because the research team was too small to monitor all entrances and exits of the clinic). Multiply-imputed data (n = 20 imputations) were used for this analysis."

Comment 5

The authors also discuss a critical deviation in the data collection in WC clinics, with 'acute care' consistently incorrectly marked by those attending for HIV care which likely resulted in inaccurate (over)reporting of HIV

treatment care (Line 191). Although it is mentioned that HIV and acute care are grouped together in the analysis, they report these groups separately both in table format and in figures which I believe is misleading to readers considering the acknowledgement of the questionable quality of the data.

Response: Thank you. As the reviewer notes, this was only an issue in Western Cape clinics, as different field teams were used in the two provinces. Western Cape numbers presented in Table 1 are accompanied by a footnote indicating that these are likely spurious, and the differentiation between 'acute' and 'HIV care' in Figure 1 is used only to illustrate the within-clinic variability in patient pathways for one clinic in KZN, where the data were considered reliable. We have made minor amendments to the text in the results section and in the footnote to table 1 to increase clarity.

Reviewer 1, comment 5, amendment 1 of 1 (Results)

Original text

"Because no identifying details of individuals were collected, this could not be rectified, and the two categories were combined in analysis (but are shown separately in Table 1)."

Amended text (page 10, lines 207–209)

"Because no identifying details of individuals were collected, this could not be rectified, and the two categories were combined for the regression analysis (but are shown separately in Table 1 for completeness)."

Comment 6

Although the dataset includes both clinics with appointment systems, and those without, and the authors discuss in detail the potential benefit of date-time appointment systems, these two systems are not compared in the analysis. Is there a particular reason for this? The results could potentially be important to having a real world comparison of the effects of appointment systems on patient flow and visit duration.

Response: Thank you for raising this important issue, which was also raised by Reviewer 2. Although many clinics in the study (particularly in Western Cape) did implement appointment systems, appointments were often given only to certain groups of patients and not others (e.g., day-time appointments for antenatal care but not for HIV care) and/or the clinic operated a 'mixed model', where some groups of attendees received date-time appointments, others received date only appointments, and others did not receive an appointment. In addition, every clinic had individuals who walked in for acute care, regardless of whether the clinic had an appointment system in place. We attempted to account for this during data collection, and our entry questionnaire (presented in Supplementary figure 2) included a question about appointments. Unfortunately this question was answered very poorly across clinics (likely due to a failure during fieldworker training and/or translation) and the data were of insufficient quality to be used.

We have added text to the limitations section to reflect this, as shown below.

Reviewer 1, comment 6, amendment 1 of 1 (Strengths and limitations)

Original text

"Future exercises should, at a minimum, continue to record clinic exits for as long as possible. Because of…"

Amended text (page 20, lines 430–432)

"Future similar exercises should, at minimum, continue to record clinic exits for as long as possible. Although we attempted to collect information on whether attendees had appointments, the data were of very low quality and we were unable to include this variable in the regression analysis. Because of…"

Reviewer #2

Major comment 1

Conceptually, it is understandable why reducing overcrowding in healthcare facilities is important to IPC measures, unfortunately, the authors fail to make an argument for why this specific study is necessary, novel, or innovative. It is not clear to me that this study was actually needed to draw the conclusions and recommendations described in this paper. Regarding specific conclusions: (1) the finding that patients who were attending clinic for TB care spent less time in clinic is not novel if there is a built-in fast track system and (2) the finding that patients designated to outdoor spaces spent more of their visit time outdoor is not novel. Again, it is not clearly stated why this study was necessary to draw these specific types of conclusions.

Response: Our thanks to the reviewer for this comment. We have addressed the relevance of this research to IPC in our response to the Editor's comment #7, above. We do not claim that our findings are entirely novel, and agree that many of them seem obvious once measured. Nevertheless, we consider this a valid exercise. South Africa has around 3,500 PHC clinics that vary in size, design, and organisation, and there are few published data that describe either the effectiveness or acceptability of complex interventions such as fast track or queueing systems or the relationships between clinic design, use of space, and patient behaviour. Regardless of the 'novelty' of our findings, our work adds to this body of knowledge and raises a number of additional questions for future research. In addition, and as we argue in the discussion (original manuscript lines 383–387), because the situation at each clinic is in constant flux, more frequent, light-touch measurement of patient flow is needed to give clinic managers guidance on where and when to intervene to reduce congestion. As stated in the introduction (original manuscript lines 51–66), one of the aims of this exercise was to develop and test a method to do this, as the approaches currently used to measure waiting times and systems efficiency can be time-consuming, with substantial delays between measurement and data availability.

We believe we have cited most of the relevant literature from South Africa, but if there are important sources that were missed, we are happy to include them. We have not made any changes to the text in response to this comment.

Major comment 2

Additionally, I am concerned by the significant amount of missing data. While, I understand that multiple imputation was used to generate arrival and/or departure times for individuals for whom the data was not recorded, why did the group not expand their daily data collection time period so that they were present to ensure that the majority of patient arrival/departure times were recorded?

Response: Thank you. We appreciate the reviewer's concerns around the amount of missing data (shared by Reviewer 1) and have added text to the 'Limitations' section of the discussion (see our response to Reviewer 1's comment #3 for details). As stated in the discussion (line 424), we were unable to extend data collection times primarily because of logistical restrictions. Our aim was to collect data from all the *Umoya omuhle* study clinics, which were spread across two provinces of South Africa. Because of constraints related to geography, time, and funds, we were forced to restrict our data collection to 11 clinics and to the times reported in the manuscript. We accept that extending data collection times would have resulted in better data around waiting times, and have made suggestions around how future exercises might approach this. However, we remain confident that our findings around the dynamics of waiting area occupancy and movement patterns within clinics are unaffected by this limitation, particularly because the data reflect the times when clinics were at their busiest.

Changes made to the text are detailed under our response to Reviewer 1's comment #3.

Additional question 1

Why did the team choose occupancy density as a proxy for patient flow? Is this a standard proxy in the field and if not, why was this chosen for this study's outcome?

Response: One of the objectives of this exercise was to find low cost methods to measure patient flow in PHC clinics. Although the movement of patients through a clinic is often thought of as linear (e.g., from entry to service 1 to service 2 to exit), this is usually not the case. Each clinic contains multiple parallel and intersecting queues of variable speeds as well as a number of physical bottlenecks and spaces where unrestricted 'mixing' takes place. We believe that one of the reasons that patient flow has received relatively little attention in the implementation of IPC is because it is difficult to measure, which in turn makes it difficult to monitor the effects of interventions to improve flow. As discussed in the manuscript (lines 400–402), previous analyses have relied heavily on observation, qualitative description, and mapping of flow – exercises that can yield useful data, but are slow and resource-intensive to conduct. There is a recognised need (expressed at our patient flow workshop by experts in South African primary care and IPC) for tools that are easier to use and that yield quick results, particularly because the situation at a clinic can change rapidly.

Room occupancy has been used by previous investigators to model the risk of respiratory pathogen transmission in prisons, health facilities, and other public spaces (see, for example, Johnstone-Robertson et

al. 2011 [PMID 22272961], Taylor et al. 2016 [PMID 27510239], and Sun & Zhai 2020 [PMID 32834937]). Calculating density (occupancy divided by room area or volume) allows for easy comparison between rooms of different sizes. We are not aware of the use of repeated estimation of occupancy density as a proxy for patient flow in these settings, but only suggest, based on our experience in this study, that it may be useful to use it in this way.

We have not made any changes to the text in response to this comment.

Additional question 2

How many days of data collection were done at each site? Was it daily for each site in KZN and WC? This is not clear in the data collection section.

Response: Thank you. There were a total 12 days of data collection at 11 clinics. Data were collected on one day from 10 clinics and on two days from one clinic (KZN1). This is described in the results section (original manuscript lines 185–187) and in the supplementary material (Supplementary tables 3 and 5).

We have not made any changes to the manuscript in response to this comment.

Additional question 3

Data was collected at different times of the year for each region. How does the time period when data was collected affect findings at each site—i.e., seasonal volume, primary chief complaints by season, weather (particularly for clinics with outdoor waiting spaces)?

Response: Thank you. We acknowledge that these are important factors that may have affected our outcomes, but we were unable to measure these variables as part of this study. We had addressed this to some extent in the original limitations (original manuscript lines 412–414) but have edited to make this more explicit, as shown below.

Reviewer 2, additional question 3, amendment 1 (Strengths and limitations)

Original text

"Because of variability between and within clinics, and because data were collected on only one day from almost all clinics, estimates presented here should not be considered representative of the two provinces, types of clinics, or the clinics themselves."

Amended text (page 20, line 432–436)

"Because of 1) the amount of missing data, 2) the variability between and within clinics, and 3) because data were collected on only one day from almost all clinics (seasonal changes in symptom/disease prevalence are also likely to affect rates of clinic attendance and therefore patient flow and waiting times), reported

estimates should not be considered representative of the two provinces, types of clinics, or the clinics themselves."

Additional question 4

Why was FIVE minutes selected for the total capture time cut off for exclusion? Are there folks that really make it in and out of clinic in less than say 10 minutes?

Response: It was observed at some clinics that a small proportion of individuals would agree to participate in the study, receive their barcode, and then immediately (or soon after) place the barcode in a pocket or bag or throw it away. The five minute cut-off was intended to screen out these individuals, and is not intended to represent a minimum length of clinic visit.

Please see our response to Reviewer 1's comment #2 for details of small changes to the text.

Additional question 5

Why does it matter that early arrival, being female, and attending with a young child lead to longer wait times? Are there significant clinical outcomes to these findings? Are there practical changes that need to be made in patient flow for these groups? This is not directly addressed.

Response: Thank you. As discussed in the limitations (original manuscript lines 412–414), because the patterns we observed may not be representative of other clinics in the country (or even of the same clinics at different times), we were reluctant to make specific recommendations based on associations seen in our analysis. In addition, because of the significant heterogeneity between clinics (size, layout, staffing, etc.) we would suggest that clinics should be making frequent assessments of waiting times and flow and using their own data to guide intervention. As discussed in the original recommendations (original manuscript lines 376–381), any changes required are likely to relate to the organisation of care and overall running of the clinic, rather than to the clinical management of individuals.

We do not think it is appropriate (or safe) to make specific recommendations beyond the broad measures already discussed, and have therefore not made any changes to the manuscript in response to this comment.

Additional question 6

If appointment systems have been shown to reduce waiting times and the study included clinics with appointment system, I would have liked to see some discussion of how the study's primary outcomes compared among clinics with appointment systems and those without. What percentage of the WC patients were part of the date-time appointment system? Was this a significant amount?

Response: Thank you - please see our response to Reviewer 1's comment #6.

Additional question 7

"In four clinics with an outdoor waiting area that was used as part of a designated patient pathway, individuals spent a median 13.7% (IQR 242 1.4–74.5; n = 1,362) of their time outdoors, compared with a median 0% (IQR 0–1.4; n = 828) outdoors among attendees at the five clinics that did not have an outdoor waiting area or where the outdoor area was not used." Why did such a small percentage of patients with a designated outdoor waiting area not wait outdoors? If patients do not spend much time in the waiting area overall, does it really matter if more clinics make more outdoor waiting space available?

Response: Thank you. Our interpretation is that the types of locations in which individuals spend their time is likely to be heavily influenced by the organisation of care and mandated 'patient pathways' at each clinic (as alluded to in the discussion – original manuscript lines 326–331). We agree with the reviewer that the overall time spent outdoors is relatively small, even in clinics with outdoor areas. However, we would contend that this should be within the power of the clinic to change, for example by altering pathways to make greater use of outdoor spaces. We have added some text to the discussion and conclusions to reflect this, as shown below.

Reviewer 2, additional question 7, amendment 1 of 2 (Discussion)

Original text

"The existence of an outdoor, 'low risk' waiting area is of little benefit if most individuals spend most of their time in poorly ventilated indoor spaces. However, the use of outdoor spaces may be less feasible in areas with lower temperatures. Thermal comfort and user acceptability are important considerations when planning changes to patient flow."

Amended text (page 16, line 343–348)

"The existence of an outdoor, 'low risk' waiting area is of little benefit if most individuals spend most of their time in poorly ventilated indoor spaces. In this study, individuals spent most of their time indoors, even in clinics with outdoor waiting areas, and this may be a missed opportunity to reduce transmission risk. However, thermal comfort and user preferences are important considerations when planning changes to patient flow, and the use of outdoor spaces may be less feasible in areas with lower temperatures (such as South Africa's Western Cape province)."

Reviewer 2, additional question 7, amendment 2 of 2 (Conclusions)

Original text

"Attendees generally waited where they were asked to: using outdoor waiting areas as part of designated patient pathways increased the proportion of visit time spent outdoors."

Amended text (page 20, line 443–445)

"Attendees generally waited where they were asked to: using outdoor waiting areas as part of designated

patient pathways increased the proportion of visit time spent outdoors, though most individuals still spent most of their time indoors, suggesting that outdoor spaces could be better utilised.”

Additional actions

We have:

1. Made a number of small additional edits to the manuscript to increase clarity (please see ‘tracked changes’ document for details);
2. Added two new references
    a. New reference #55: Govender et al. *Clin Infect Dis* 2021
    b. New reference #56: Nathavitharana et al. *Int J Tuberc Lung Dis* 2017
3. Uploaded ‘tracked changes’ and ‘clean’ versions of the revised manuscript to Editorial Manager; and
4. Uploaded a ‘clean’ version of the revised supplementary material to Editorial Manager.

Thank you again for your review of this article. We trust that these revisions are satisfactory and look forward to hearing from you.

Yours sincerely,

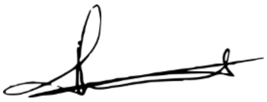

Aaron Karat
Corresponding author
